# Chimeric Antigen Receptor T-Cell Therapy in Paediatric B-Cell Precursor Acute Lymphoblastic Leukaemia: Curative Treatment Option or Bridge to Transplant?

**DOI:** 10.3389/fped.2021.784024

**Published:** 2022-01-25

**Authors:** Jochen Buechner, Ignazio Caruana, Annette Künkele, Susana Rives, Kim Vettenranta, Peter Bader, Christina Peters, André Baruchel, Friso G. Calkoen

**Affiliations:** ^1^Department of Pediatric Hematology and Oncology, Oslo University Hospital, Oslo, Norway; ^2^Department of Paediatric Haematology, Oncology and Stem Cell Transplantation, University Hospital Würzburg, Würzburg, Germany; ^3^Department of Pediatric Oncology and Hematology, Charité—Universitätsmedizin Berlin, Corporate Member of Freie Universität Berlin and Humboldt-Universität zu Berlin, Berlin, Germany; ^4^Department of Pediatric Hematology and Oncology, Hospital Sant Joan de Déu de Barcelona, Institut per la Recerca Sant Joan de Déu, Barcelona, Spain; ^5^University of Helsinki and Children's Hospital, University of Helsinki, Helsinki, Finland; ^6^Division for Stem Cell Transplantation, Immunology and Intensive Care Medicine, Department for Children and Adolescents, University Hospital, Goethe University, Frankfurt, Germany; ^7^St. Anna Children's Hospital, Medical University Vienna, Vienna, Austria; ^8^St. Anna Children's Cancer Research Institute, Vienna, Austria; ^9^Université de Paris et Institut de Recherche Saint-Louis (EA 35-18) and Hôpital Universitaire Robert Debré (APHP), Paris, France; ^10^Department of Stem Cell Transplantation and Cellular Therapy, Princess Máxima Center for Pediatric Oncology, Utrecht, Netherlands

**Keywords:** CAR (chimeric antigen receptor) T cells, child, haematopoietic stem cell transplantation, ALL (acute lymphoblastic leukaemia), B-ALL, bridge to allogeneic stem cell transplantation, curative therapy

## Abstract

Chimeric antigen receptor T-cell therapy (CAR-T) targeting CD19 has been associated with remarkable responses in paediatric patients and adolescents and young adults (AYA) with relapsed/refractory (R/R) B-cell precursor acute lymphoblastic leukaemia (BCP-ALL). Tisagenlecleucel, the first approved CD19 CAR-T, has become a viable treatment option for paediatric patients and AYAs with BCP-ALL relapsing repeatedly or after haematopoietic stem cell transplantation (HSCT). Based on the chimeric antigen receptor molecular design and the presence of a 4-1BB costimulatory domain, tisagenlecleucel can persist for a long time and thereby provide sustained leukaemia control. “Real-world” experience with tisagenlecleucel confirms the safety and efficacy profile observed in the pivotal registration trial. Recent guidelines for the recognition, management and prevention of the two most common adverse events related to CAR-T — cytokine release syndrome and immune-cell–associated neurotoxicity syndrome — have helped to further decrease treatment toxicity. Consequently, the questions of how and for whom CD19 CAR-T could substitute HSCT in BCP-ALL are inevitable. Currently, 40–50% of R/R BCP-ALL patients relapse post CD19 CAR-T with either CD19^−^ or CD19^+^ disease, and consolidative HSCT has been proposed to avoid disease recurrence. Contrarily, CD19 CAR-T is currently being investigated in the upfront treatment of high-risk BCP-ALL with an aim to avoid allogeneic HSCT and associated treatment-related morbidity, mortality and late effects. To improve survival and decrease long-term side effects in children with BCP-ALL, it is important to define parameters predicting the success or failure of CAR-T, allowing the careful selection of candidates in need of HSCT consolidation. In this review, we describe the current clinical evidence on CAR-T in BCP-ALL and discuss factors associated with response to or failure of this therapy: product specifications, patient- and disease-related factors and the impact of additional therapies given before (e.g., blinatumomab and inotuzumab ozogamicin) or after infusion (e.g., CAR-T re-infusion and/or checkpoint inhibition). We discuss where to position CAR-T in the treatment of BCP-ALL and present considerations for the design of supportive trials for the different phases of disease. Finally, we elaborate on clinical settings in which CAR-T might indeed replace HSCT.

## Introduction

Outcomes among paediatric patients and adolescents and young adults (AYAs) with B-cell precursor acute lymphoblastic leukaemia (BCP-ALL) have continuously improved in recent decades, with long-term survival rates now reaching 90% in children and 70% in young adults treated on contemporary protocols (1–3). However, 15–20% of paediatric patients and almost 30–40% of young adult patients relapse or remain refractory to primary therapy (4). Outcomes for patients who experience early bone marrow relapse (<18 months), have ≥2 relapses, a relapse after allogeneic haematopoietic stem cell transplantation (HSCT) or who are refractory to induction therapy are historically very poor (5, 6). Until recently, the standard of care for these relapsed/refractory (R/R) patients was based on intensive block chemotherapy followed by consolidation with HSCT if deep remission could be achieved.

In the last decade, however, the advent of targeted immunotherapies, e.g., the bispecific antibody blinatumomab (anti-CD19/anti-CD3), the antibody-drug conjugate inotuzumab ozogamicin (anti-CD22) and chimeric antigen receptor (CAR) T-cell therapy (herein referred to as CAR-T for brevity) has provided novel tools to achieve responses in patients with resistant leukaemia and dramatically augmented treatment options for R/R BCP-ALL (7).

**Figure 1:**
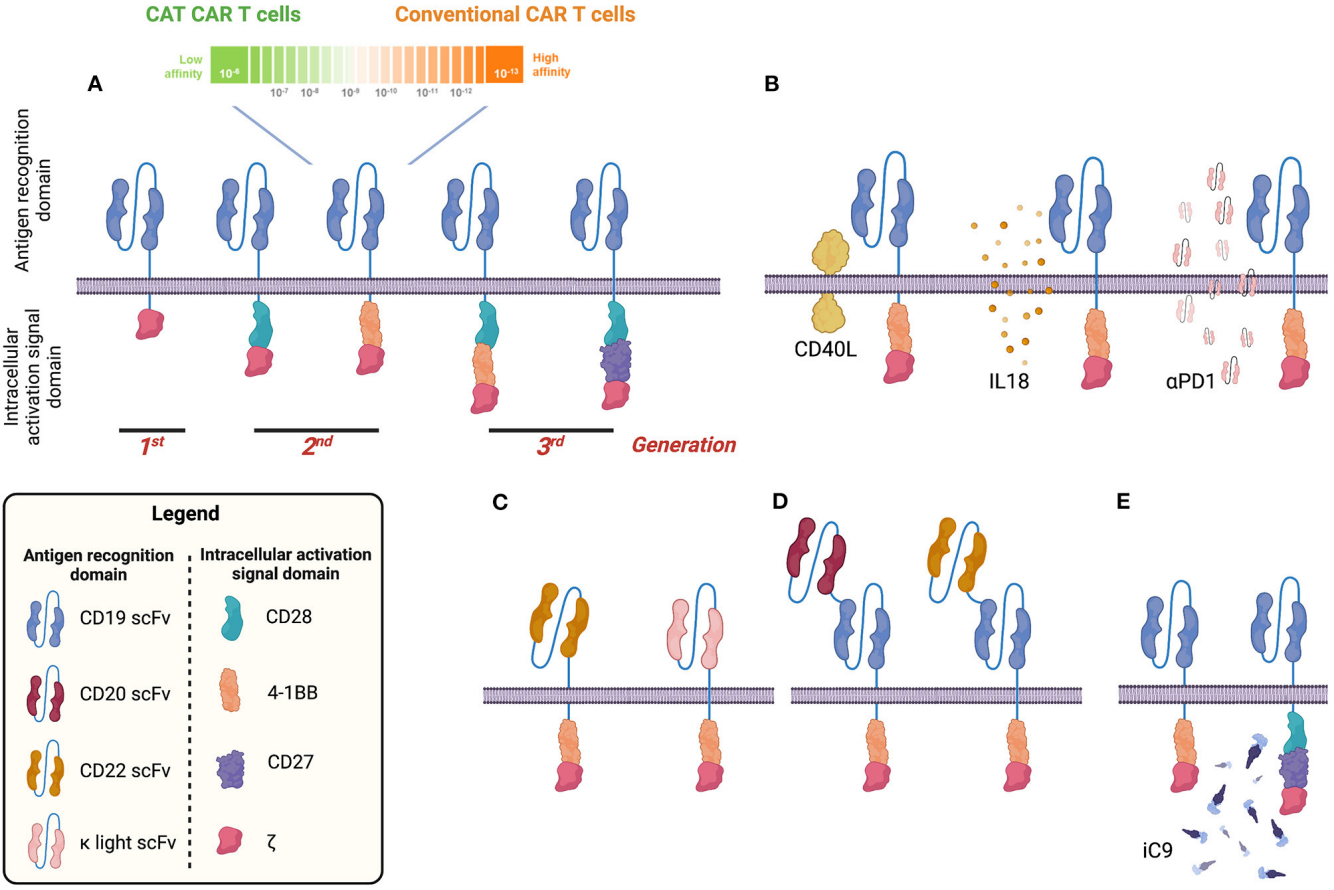
Visual summary of different chimeric antigen receptor (CAR) designs to target B-cell malignancies. CD19 CAR has been tested in many clinical trials so far. Different generations of CD19 CAR have been developed including a second-generation CD19 CAR with low affinity for the CD19 antigen (CAT CAR) **(A)**. Several groups proposed strategies to improve the long-term efficacy of the CD19 CAR by armoured CAR constructs capable of expressing both CD19 CAR and other molecules such as CD40L, interleukin 18 (IL-18), or programmed death 1 (PD1) capable of improving cytotoxic activity, reducing the exhaustion profile and sustaining the proliferation and persistence of CAR T cells **(B)**. In addition to CD19, other B-cell antigens have been investigated and CARs have been generated and tested in preclinical and early-stage human clinical studies (e.g., CD22 and κ light chain) **(C)**. To avoid tumour escape, bispecific CARs have been developed targeting, for example, CD19 and CD20 or CD22 **(D)**. To improve the safety profile and generate a tool to mitigate/abrogate side effects, a suicide gene based on an inducible caspase 9 (iC9) has been developed and validated **(E)**. Image created with BioRender.com.

A CAR combines an antigen recognition domain [typically a single-chain variable fragment (scFv) of a monoclonal antibody] with intracellular activation signal domains of immune-effector T cells (Figure 1A) (8, 9). The addition of a costimulatory domain (e.g., 4-1BB, CD28, or OX40) in second-generation CARs or two costimulatory domains (CD28.4-1BB) in third-generation CARs provides clinically meaningful activity and persistence of CAR T cells (10, 11) (Figure 1A). Because of such properties, CAR-T is being investigated as a potential stand-alone treatment in R/R BCP-ALL.

Based on the results of the pivotal ELIANA trial (ClinicalTrials.gov identifier: NCT02435849) (12) and subsequent approval of tisagenlecleucel, an anti-CD19 CAR-T product, for the treatment of CD19^+^ R/R BCP-ALL by the US Food and Drug Administration (FDA) and European Medicines Agency (EMA) in 2017/18, a rapidly increasing number of paediatric stem cell transplantation centres have been certified to administer tisagenlecleucel to paediatric patients and AYAs, and hundreds of products have been infused worldwide as a novel standard-of-care treatment option. Currently, the three main clinical scenarios in which physicians offer tisagenlecleucel to BCP-ALL patients are: (1) when an HSCT was previously performed but failed to be efficacious (post-HSCT relapse); (2) in chemotherapy-resistant ALL patients ineligible for HSCT because minimal residual disease (MRD) remission cannot be achieved (refractory patients); or (3) a belief and hope that CAR-T is as effective as but less toxic than HSCT to eradicate the resistant leukaemic clone and, therefore, is favoured over HSCT (patients with ≥2 relapses who have not been transplanted before and are, *per se*, eligible for HSCT). In exceptional cases, depending on national regulations and the doctor's degree of conviction, the indication to use commercial tisagenlecleucel might be more liberal, for example by stretching the definition of refractory disease to patients not achieving MRD-negativity at certain treatment time points after first relapse. The international multicentre CASSIOPEIA study (NCT03876769) is the only active study evaluating this approach in primary BCP-ALL for National Cancer Institute (NCI)-defined high-risk patients with MRD at end of consolidation.

However, randomised studies directly and prospectively comparing HSCT and CAR-T efficacy outcomes have not yet been performed in paediatric ALL, and the longest follow-up post CAR-T to date is in a patient infused with tisagenlecleucel <10 years ago (13). Tisagenlecleucel brings considerable costs to healthcare systems but is cost-effective if given as definitive treatment for long-term cure (14, 15). However, 40–50% of patients initially responding to tisagenlecleucel relapse; a proportion of patients receive HSCT additionally; and 10–20% are primary refractory to tisagenlecleucel. In other trials with different CAR-T products, all responding patients were allocated to HSCT consolidation (16–18). Therefore, central and elusive questions are the extent to which CAR-T is a stand-alone curative treatment, particularly with longer follow-up, and whether patients need additional HSCT either as consolidation for remission or treatment of relapse post CAR-T.

Of note, the international ALL SCTped 2012 For Omitting Radiation Under Majority age (FORUM) trial (NCT01949129) recently reported, among patients being in complete remission (CR) 1–3, an excellent 2-year overall survival (OS) rate of 91% and cumulative incidence of relapse (CIR) and treatment-related mortality of 12 and 2%, respectively, if HSCT was performed uniformly using a standardised protocol of total body irradiation (TBI) plus etoposide (19). However, late effects after TBI conditioning remain of great concern (20, 21).

In this review, we summarise the current data on tisagenlecleucel and other CAR-T products in paediatric BCP-ALL, focusing on: (1) the fraction of patients receiving HSCT or other post-infusion interventions, either prophylactically, pre-emptively or for relapse post infusion; and (2) reported factors that influence the efficacy and long-term performance of CAR-T, including CAR design and pre-infusion therapies, to identify evidence that might guide decisions regarding if and when consolidative HSCT should be performed. Finally, we define knowledge gaps and propose necessary studies to better clarify where to place CAR-T in the overall treatment concept to cure paediatric ALL, with a focus on minimising late treatment-related side effects.

## Summary of Major Clinical Trials EvaluatinG CAR-T in Paediatric R/R BCP-ALL

The first two children who received CD19 CAR-T for R/R BCP-ALL were reported in 2013 (13). In the following 8 years, a body of evidence has grown on the efficacy and safety of CAR-T in paediatric patients and AYAs with ALL, primarily targeting CD19, but also CD22 and other (or combined) antigens. Below and in Table 1 we summarise the main clinical studies performed with CD19- and CD22-specific CAR-T products, focusing on efficacy outcomes, CAR T-cell persistence and post-infusion interventions (12, 16–18, 22–30).

**Table 1:** Overview of the main clinical studies investigating CD19- or CD22-targeted CAR-T in paediatric patients and AYA with BCP-ALL.

**Research group, trial phase**	**References**	**Patients, *N***	**Prior HSCT, *n* (%)**	**Overall response rate (within 3 months)**	**Efficacy (beyond 3 months)**	**CAR-T cell persistence**	ALL-targeted interventions post CART^*^
**Tisagenlecleucel, phase I/II studies**							
CHOP, I/IIa	(22)	30 (25 P+AYA)	18 (60%)	90% (27/30) at 1 mo.	6-mo. EFS: 67% 6-mo. OS: 78%	6-mo. persistence: 68%	3 HSCT (11%^†^), 1 DLI, 1 re-infusion
CHOP, I/IIa	(23)	59 (P+AYA)	39 (61%)	93% (55/59) at 1 mo.	6-mo. RFS: 76% 12-mo. RFS: 55% 12-mo. OS: 79%	Unknown	5 HSCT (9%^†^), 1 DLI, 17 re-infusion
ELIANA, II	(12)	75 (P+AYA)	46 (61%)	81% (61/75) at 3 mo.	6-mo. EFS: 73% 12-mo. EFS: 50% 12-mo. OS: 76%	6-mo persistence: 83% Median persistence: 168 days	8 HSCT (13%^†^), 4 huCART19, 1 ponatinib, 1 vincristine and blinatumomab, 1 ATG
**Tisagenlecleucel, real-world experience**							
CIBMTR, retrospective	(24)	255 (0.4–26 yrs)	71 (28%)	86% (213/249) at 3 mo.	6-mo DoR: 78% 6-mo OS: 89%	Unknown	34 HSCT (16%^†^)
**CD19 CAR-T other than tisagenlecleucel**							
CARPALL, II	(25)	14 (P+AYA)	10 (71%)	86% (12/14) at 3 mo.	1-yr EFS: 46% 1-yr OS: 63%	Median persistence: 215 days	0 HSCT
Seattle, I	(26)	45 (P)	28 (62%)	93% (40/43) at Day 21	12-mo. EFS: 50% 12-mo. OS: 66%	Median duration: 3 mo.	11 HSCT (28%^†^), 10 re-infusions
NCI, I	(18, 27)	20 (P+AYA)	7 (35%)	70% (14/20) at Day 28	OS: 52% (Median FU 10 mo.)	Maximum persistence: 68 days	10 HSCT (71%^†^), 3 re-infusions
MSKCC, I	(16)	25 (P+AYA)	18 (75%)	75% (18/24) at Day 28	Dependent on LD/ cell dose	Median persistence: 7 days	15 HSCT (83%^†^)
Sheba, Ib/II	(17)	20 (18 P+AYA)	10 (50%)	90% (18/20) at Day 28	1-yr EFS: 73% 1-yr OS: 90%	Median persistence 23 days	14 HSCT (77%^†^)
Barcelona, I	(28)	38 (19 P+AYA)	33 (87%)	84% (32/38) at Day 28	P: 1-yr DFS 82% P: 1-yr OS 78%	P: BCA at 1 yr: 48%	NR
**CD22 CAR-T**							
NCI, I	(29)	21 (P+AYA)	21 (100%)	Dependent on cell dose	Relapse: 8/12 responders	Maximum persistence: 18 mo.	None
NCI, I	(30)	58 (55 ALL P+AYA)	39 (67%)	72% (40/55 ALL) at Day 28	Median OS: 13.4 mo. Median RFS: 6.0 mo.	Unknown	13 HSCT (33%^†^; 100% of MRD-negative patients), 1 re-infusion

* *While still in CR*.

† *Out of those patients who responded (CR)*.

*ATG, anti-thymocyte globulin; AYA, adolescent and young adult; BCA, B-cell aplasia; BCP-ALL, B-cell precursor acute lymphoblastic leukaemia; CAR-T, chimeric antigen receptor T-cell therapy; CHOP, Children's Hospital of Philadelphia; CIBMTR, Centre for International Blood and Marrow Transplant Research; DLI, donor lymphocyte infusion; DoR, duration of response; DFS, disease-free survival; EFS, event-free survival; FU, Follow-up; HSCT, haematopoietic stem cell transplantation; huCART19, human CD19 CAR-T; LD, Lymphodepletion; LFS, leukaemia-free survival; MSKCC, Memorial Sloan Kettering Cancer Centre; mo., month; OS, overall survival; NCI, National Cancer Institute; NR, not reported; P, paediatric; RFS, relapse-free survival; yr, year*.

### Tisagenlecleucel

Tisagenlecleucel, formerly known as CTL019 and now commercialised as KYMRIAH®, is an autologous, second-generation anti-CD19 CAR-T developed by Novartis Pharmaceuticals in collaboration with the University of Pennsylvania and Children's Hospital of Philadelphia (CHOP). It contains a CAR composed of an anti-CD19 scFv (from the recombinant monoclonal murine antibody clone FMC63) for CD19 antigen recognition, a CD8-α hinge region, a 4-1BB (CD137) costimulatory domain and CD3ζ as a signalling domain (31). It utilises lentivirus for T-cell transduction.

#### Phase I/II Trials of Tisagenlecleucel in Paediatric R/R BCP-ALL

The first trial investigating tisagenlecleucel in paediatric CD19^+^ R/R BCP-ALL was a Phase I/IIa single-arm study at the CHOP (NCT01626495 and NCT01029366). The initial publication reported outcomes in 30 patients (including 25 paediatric ALL patients aged 5–22 years at infusion) of whom 18 had relapsed after previous HSCT (22). The overall remission rate (ORR), including CR and CR with an incomplete haematologic recovery (CRi), at 1 month after infusion was 90%; 22 of the 25 evaluable patients were MRD-negative as assessed by flow cytometry (FCM). Of the 27 patients who achieved CR, 19 remained in remission at a median follow-up of 7 months. Fifteen patients received no further ALL-targeted therapy, while five (19% of all the responders) were allocated to additional post-infusion interventions: three underwent HSCT while in remission; one received bortezomib and an infusion of donor lymphocytes for MRD reappearance; and one received tisagenlecleucel re-infusions due to an early re-appearance of B cells as a marker for CAR T-cell loss. The probability of persistence of tisagenlecleucel at 6 months for all infused patients was 68%.

In an update from the same study with longer follow-up, 59 paediatric patients (aged 20 months to 24 years) including 39 patients with a relapse post HSCT were reported on (23). Fifty-five patients (93%) were in CR/CRi 1 month post infusion and 52 were MRD-negative by FCM. Five of 59 patients (8% of all responders) were consolidated by HSCT. Of note, 17 of the 55 responders (31%) received tisagenlecleucel re-infusions 3 and/or 6 months post initial infusion because of the reappearance of CD19^+^ MRD (three patients), B-cell recovery (seven patients), or appearance of CD19^+^ haematogones in the bone marrow (seven patients) (32).

In the Phase II ENSIGN trial (NCT02228096), the safety and efficacy of tisagenlecleucel was for the first time investigated in a multicentre setting at 13 US sites with centralised manufacturing of all products at the University of Pennsylvania. ENSIGN was instrumental in transferring manufacturing from a single-centre academic setting to an industry-based manufacturer (Novartis) and laid the foundation for the global ELIANA trial. ENSIGN enrolled 73 patients of whom 58 had been infused at last available study report (33). The ORR (CR+CRi) within 6 months of infusion was 69%. Tisagenlecleucel was detected in the peripheral blood for up to 764 days in responders.

The global ELIANA Phase II registration trial (NCT02435849) investigated the safety and efficacy of tisagenlecleucel in paediatric patients with R/R BCP-ALL at 25 study sites in 11 countries across North America, Europe, Asia and Australia. In the primary publication, 75 infused patients (aged 3–23 years at enrolment) were reported on, of whom 61% had relapsed after prior HSCT (12). Sixty-one patients (81%) were in CR/CRi within 3 months post infusion. In total, 15 of the responders (25%) received additional ALL-targeted therapies post infusion: eight (13%) underwent HSCT (two due to early loss of B-cell aplasia [BCA], two due to MRD in the bone marrow, and four for unknown reasons), seven (12%) received new cancer therapies other than HSCT during morphological remission [four humanised CD19 CAR-T, one ponatinib, one vincristine and blinatumomab, and one anti-thymocyte globulin (ATG)].

In the latest published update from ELIANA (34), 79 patients had been infused with a median follow-up of 24 months (range, 4.5–35 months). The ORR within 3 months was 82% (65/79 patients), and relapse-free survival rate among responders was 59% at 2 years. Nineteen patients relapsed post infusion, 14 of them with CD19^−^ disease.

Finally, in the tisagenlecleucel Phase IIIb expanded-access study (CCTL019B2001X, NCT03123939), an ELIANA confirmatory trial specifically focusing on pre-infusion exposure to blinatumomab and inotuzumab ozogamicin, 67 paediatric and AYA ALL patients (aged 3–33 years at enrolment) received tisagenlecleucel (35). Fifteen patients received blinatumomab and nine received inotuzumab ozogamicin at any time point before tisagenlecleucel [with a time from last dose of blinatumomab or inotuzumab ozogamicin to infusion of a median 372 days (range, 130–932) and 86 days (range 32–172), respectively]. The ORR at 3 months was 85% for the whole cohort, confirming the ELIANA experience. However, the ORR was only 67% for patients who had previously received blinatumomab or inotuzumab ozogamicin. In total, 14 patients relapsed: nine with CD19^−^ disease (two after blinatumomab) and five with CD19^+^ disease (three after inotuzumab ozogamicin). Of note, patients who had received prior blinatumomab or inotuzumab ozogamicin as bridging therapy had a 12-month OS rate of 53 and 71%, respectively, compared with 83% in patients without previous exposure to either drug. Although patient numbers were too low to draw definite conclusions, and the use of inotuzumab ozogamicin or blinatumomab might reflect a subgroup of patients with particularly resistant disease, pre-treatment with inotuzumab ozogamicin seemed to affect expansion (C_max_), persistence (T_last_) and thereby the total area under the curve (AUC)_0−28*d*_ of tisagenlecleucel. The number of patients who underwent HSCT or other post-infusion interventions were not reported.

To summarise, in clinical trials with tisagenlecleucel for paediatric R/R BCP-ALL, a fraction of patients were cured by a single-infusion of tisagenlecleucel, even after multiple previous lines of therapy and without post-infusion intervention. About 10–15% of patients who initially responded to CAR-T infusions later received consolidative HSCT while in remission. Reported indications to proceed to HSCT were either a lack of CAR T-cell persistence or early loss of BCA with the aim to prevent a CD19^+^ relapse (consolidation or re-appearance/persistence of MRD post-infusion, i.e., pre-emptive therapy). However, published data leave uncertainty on the total number of patients having undergone transplantation post tisagenlecleucel, as some patients were transplanted due to frank relapses and therefore censored in the analyses at the time of relapse. Such patients were only followed for survival but subsequent therapies including HSCT might not have been captured and reported. A smaller proportion of patients received repeated infusions of tisagenlecleucel with the aim to prevent relapse. The role of re-infusions in the overall outcome after tisagenlecleucel therapy cannot be retrieved from published reports. How often additional infusion bags were available and how patients were selected to receive re-infusions were not reported in detail.

#### Tisagenlecleucel for Paediatric R/R BCP-ALL Outside the Clinical Trial Setting

**Figure 2:**
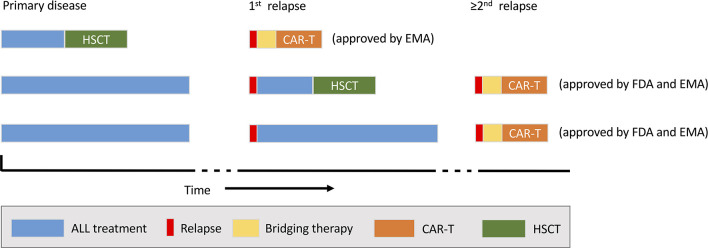
Current indications for commercial chimeric antigen receptor (CAR) T-cell therapy (tisagenlecleucel). The possible timing of CAR-T (orange) within the treatment sequence for acute lymphoblast leukaemia (ALL) and relative to haematopoietic stem cell transplantation (HSCT; blue) is shown. EMA, European Medicines Agency; FDA, US Food and Drug Administration.

Tisagenlecleucel was approved by FDA in 2017 and EMA in 2018. Approved indications are a second or higher relapse or refractory disease of CD19^+^ BCP-ALL in patients ≤ 25 years of age (US and Europe) and any relapse post HSCT (Europe only) (Figure 2). Since marketing approval, rapidly increasing numbers of patients have been infused with tisagenlecleucel for these indications and substantial “real-world” experience has emerged from patient cohorts treated with commercial KYMRIAH® (24, 36, 37).

The largest reported cohort so far originated from the prospective, multicentre, observational Centre for International Blood and Marrow Transplant Research (CIBMTR) study conducted at 130 centres in the US and Canada. By the end of January 2020, the efficacy and safety outcomes of 255 infused paediatric and AYA R/R BCP-ALL patients (median age 13.5 years, range 0.4–26 years) were collected (24). Twenty-eight percent of the patients had relapsed after a prior HSCT, which is substantially lower than the 61% in the ELIANA cohort and likely indicate a less advanced patient cohort compared with ELIANA (12). Of note, 15 and 11% of patients had received blinatumomab or inotuzumab ozogamicin at some time prior to tisagenlecleucel infusion. The median percentage of bone marrow blasts directly before infusion was 2% (range, 0–100%); one-third of patients had >5% marrow blasts, with a median blast percentage of 48% (range, 6–100%). The ORR was 86%, comparable to that in ELIANA (12). Among patients who achieved CR, 34 (16%) went on to undergo HSCT while in remission for consolidation; an additional 21 patients were transplanted for disease relapse. In the subgroups of patients who received prior treatment with blinatumomab (*n* = 37) or inotuzumab ozogamicin (*n* = 26), the CRR was 78 and 65%, respectively. Of note, 46 and 62% of patients who received blinatumomab or inotuzumab ozogamicin, respectively, experienced treatment failure, relapse and/or died of ALL during a median of 10.9 months' follow-up.

### CAT CAR-T (Low-Affinity Anti-CD19 CAR T Cells)

With the goal to further improve the efficacy and prolong the persistence of CD19 CAR T cells by modulating the binding affinity to the cognate antigen, a CD19 scFv termed “CAT” was developed at University College London/Great Ormond Street Hospital for Children, UK, with a substantially (>40-fold) lower affinity to CD19 than the FMC63 scFv (25). This CD19 low-affinity CAR with an otherwise similar structure to tisagenlecleucel (CD8-derived stalk/transmembrane region, 4-1BB costimulatory domain and CD3ζ chain) showed faster dissociation from CD19 than FMC63. T cells expressing the CAT CAR showed enhanced cytotoxic and proliferative responses *in vitro* compared to the FMC63 CAR, possibly caused by serial T-cell triggering due to a shorter receptor–ligand interaction with enhanced signalling through proliferative pathways, decreased apoptosis and interleukin (IL)-7 signalling (25). The safety and efficacy of CAT CAR-T were subsequently investigated in the Phase II CARPALL study (NCT02443831) in which 14 R/R BCP-ALL patients were infused, 10 (71%) after post-HSCT relapse (25). By 3 months, 12 patients (86%) had achieved molecular CR. At a median follow-up of 14 months, five patients (36%) were alive and disease free. Using event-free survival (EFS) criteria in which a molecular relapse was defined as an event, EFS was 55 and 31% at 6 and 12 months, respectively. Importantly, no infused patient underwent consolidative HSCT or re-infusion. CAT CAR T cells were detectable by quantitative polymerase chain reaction (qPCR) in 11 patients (79%) at last follow-up, which was 24 months post infusion in two patients. The median duration of persistence of CAT CAR T cells at data cut-off was 215 days (range, 14–728 days). Although the CAT CAR design indeed resulted in a prolonged median half-life of the CAR T cells (34 days) compared to tisagenlecleucel [median half-life in responders: 16.8 days (38)], the efficacy was comparable between the two products. Of note, 10 of the 14 patients infused with CAT CAR-T had low-level disease (six with MRD-positive disease and four with MRD-negative disease at the time of lymphodepletion), whereas the major cause of treatment failure was CD19^−^ relapse, which occurred particularly in patients with a higher tumour burden.

### Other Anti-CD19 CAR-T Products With a 4-1BB Costimulatory Domain

The Seattle group designed a CAR-T product consisting of CD19(FCM63).CD28(transmembrane domain).4-1BB.CD3ζ transduced autologous CD4^+^ and CD8^+^ T cells using a lentivirus platform. These cells were infused in a defined 1:1 CD4^+^:CD8^+^ CAR T-cell ratio (39). In the PLAT-02 Phase I study (NCT02028455), 43 of 45 enrolled patients with R/R BCP-ALL (median age 12.3 years, range 1.3–25.4) (26) were infused with the CAR-T product, 28 (62%) for post-HSCT relapse. Seven patients had previously received a CD19-directed therapy: blinatumomab (*n* = 6) or second-generation CD19-specific CAR T cells with CD28ζ as the costimulatory molecule (*n* = 1). The rate of MRD-negative CR by FCM on day 28 was 93%. At a median follow-up of 9.6 months, 18 of the 40 patients who achieved CR subsequently relapsed. Median duration of BCA as a surrogate marker for CAR T-cell persistence was 3 months. A risk factor for relapse with CD19^+^ disease was a shorter duration of BCA. Eleven patients (28% of responders) underwent consolidative HSCT. Of the 29 patients not transplanted, 13 remained in remission while 16 patients (55%) relapsed. Factors predicting the persistence of BCA were pre-infusion CD19^+^ antigen load (blast count and/or normal B cells in the bone marrow) of >15% (median persistence 6.4 months, compared with 1.7 months for patients with a load of <15%), and the use of lymphodepleting chemotherapy before infusion. Of note, 10 patients received CAR-T re-infusions: eight due to loss of BCA (two of them re-engrafted CAR T cells) and two due to MRD persistence/reappearance; however, no anti-leukaemic effect was observed.

At the Hospital Clínic in Barcelona, a CD19 CAR-T termed ARI-0001 was developed to generate affordable CAR-T in academic institutions. The CAR consists of an scFv derived from the A3B1 antibody, a CD8 hinge and transmembrane region, 4-1BB and CD3ζ. A lentiviral vector and the CliniMACS® Prodigy device were used as the cell production platform. In the CART19-BE-01 trial (NCT03144583)—one of the first European academic clinical trials of CD19 CAR-T−47 patients with B-cell malignancies were infused with ARI-0001, among them 38 with R/R BCP-ALL (including 11 children) of whom 87% had post-HSCT relapse (28). The MRD-negative CR rate was 84%. Focusing on the paediatric patients, the 1-year PFS and 1-year OS were 82 and 78%, respectively, and 1-year probability of BCA was 48%. No patient underwent consolidative HSCT. Re-infusions were given to six patients, either for relapse or loss of BCA, without clinically meaningful or sustained efficacy.

Studies directly or prospectively comparing the 4-1BB CAR-T products developed at Seattle and Barcelona and tisagenlecleucel have not yet been performed.

### Anti-CD19 CAR-T Products With a CD28 Costimulatory Domain

At the NCI, a CAR with a CD28 costimulatory domain (CD19.28ζ CAR) was developed (40). This consists of an anti-CD19 scFv derived from FMC63, a portion of the human CD28 molecule as the transmembrane and costimulatory domain, and CD3ζ as the intracellular signalling domain. Utilising γ-retrovirus for the transduction of autologous T cells, it was clinically tested in a Phase I study (NCT01593696) in which 20 patients with BCP-ALL aged 4–27 years were infused (18). Fourteen patients (70% of all enrolled and infused BCP-ALL patients, intent-to-treat) responded with CR at day 28; 12 were also MRD-negative. All responders were per protocol candidates for consolidative HSCT. Ten patients underwent HSCT (median time to HSCT, 51 days) while in CAR-induced MRD-negative remission. All remained disease free. Two patients were judged ineligible for HSCT; both relapsed with CD19^−^ leukaemia at 3 and 5 months. The rate of leukaemia-free survival in the 12 patients who achieved MRD-negative CR was 79%. The OS at a median follow-up of 10 months was 52%. Three patients received second infusions of CD19.28ζ CAR T-cells for residual or recurrent BCP-ALL; none had objective responses. Thirteen responders had signs of B-cell recovery by day 28 as a marker for CAR T-cell contraction. No CAR T cells were detected beyond day 68 in any patient. In the recent long-term report on 50 infused paediatric and AYA patients with a median follow-up of 4.8 years (27), 28 (56%) achieved MRD-negative CR at day 28. Of these, 21 proceeded to HSCT, of whom two relapsed. The 5-year EFS post HSCT, however, was 62%, with most events attributable to treatment-related mortality. The trial demonstrated that sequential therapy with CD19.28ζ-CAR T cells and HSCT in responding patients can mediate durable disease control.

At the Memorial Sloan Kettering Cancer Centre, another second-generation CD28-based CAR was developed (termed 19-28ζ); this gene is retrovirally transduced into autologous T cells and infused after lymphodepletion with cyclophosphamide alone (41). In a paediatric and AYA Phase I study (NCT01860937), 25 patients with a median age of 13.5 years (range, 1–22.5) were infused, five for post-HSCT relapse (16). The overall CR rate at day 28 was 75% (18 of 24 evaluable patients) with 16 (89%) being MRD-negative. All 18 responders were per protocol candidates for consolidative HSCT and 15 (83%) underwent HSCT. With a median follow-up of 8 months (29 months in responders), eight patients (53%) were alive and disease free after CAR-T consolidated by HSCT; the three responders who did not undergo HSCT relapsed and died.

Finally, in a report from the Sheba Medical Centre in Israel, CAR T cells with a FMC63-derived scFv, a CD28 costimulatory domain and a CD3ζ signalling domain were produced in-house and infused into 21 patients after lymphodepletion with fludarabine and cyclophosphamide (Phase Ib/II study, NCT02772198) (17). Median age was 11 years (range, 5–48), and 10 patients had relapsed after prior HSCT. All responding patients were per protocol candidates for consolidative HSCT, irrespective of previous HSCT. Eighteen of the 20 patients (90%) who survived until day 28 after CAR-T infusion were in CR; 11 of the 14 evaluable patients were MRD-negative. The median persistence of CAR T cells (measured by qPCR in peripheral blood) was 23 days. Fourteen of the 18 responders underwent consolidative HSCT. With a median follow-up of 9 months from cell infusion, 14 patients were alive and disease free, 12 had received HSCT, and two were not transplanted. The estimated 1-year EFS was 73% and OS was 90%.

### CD22-Targeted CAR-T Products

The NCI and other groups have developed CARs targeting CD22 as an alternative antigen in BCP-ALL patients not responding to or relapsing after CD19-targeted strategies, particularly those with CD19^−^ disease. In a Phase I trial at the NCI (NCT02315612), a CAR containing a fully humanised anti-CD22 scFv region, a CD8α transmembrane domain, a 4-1BB costimulatory domain and CD3ζ (CD22.BB.z) was transduced by lentivirus into autologous T cells. Twenty-one patients were infused; the median age was 19 years (range, 7–30 years) (29). Importantly, all patients had undergone ≥1 prior HSCT; 17 had received prior CD19-directed immunotherapies, including 15 who had received CD19 CAR-T; and 10 patients had CD19^−^ or CD19-diminished disease. The CD22 CAR T cells were detectable in the blood of 19 of 21 infused patients, peaking on day 14 and remaining detectable in seven of nine patients evaluated 3 months post infusion, in two of three patients evaluated at 6 months, one patient evaluated at 9 months, and one patient evaluated at 18 months. Twelve patients (57%) achieved CR and nine were MRD-negative. Responses varied by cell dose infused, with response rates comparable to the results reported with CD19 CAR-T when the recommended Phase II dose was applied [11 of 15 (73%) patients achieved CR]. Most importantly, there was no evidence that previous CD19-directed immunotherapy or diminished surface expression of CD19 impacted on the response to CD22 CAR-T. However, eight of the 12 responders relapsed 1.5–12 months (median, 6 months) post CD22 CAR-T infusion, and relapses in seven patients followed diminished CD22 surface expression and site density, most probably due to post-transcriptional changes in CD22 protein levels.

In an update from the same trial (30), the manufacturing process was refined to include CD4^+^/CD8^+^ T-cell selection of all starting apheresis material and an adjustment of the dose to lower levels because of increased inflammatory responses caused by selection. Fifty-eight infused patients (median age 17.5 years, range 4.4–30.6 years) were reported on, of whom 55 had BCP-ALL and were evaluable for response. Forty patients (73%) achieved CR and 35 (64%) were MRD-negative by FCM. Patients who had received prior CD22-targeted therapy (either inotuzumab ozogamicin or a CD22 CAR-T) had lower MRD-negative CR rates, and 50% of these patients relapsed with CD22-diminished/negative disease. Thirteen patients proceeded to HSCT, including all who had achieved MRD-negative CR and had not been transplanted before (except one patient with intracranial haemorrhage). Median time from CAR-T infusion to HSCT was 72 days (range, 49–126 days). Overall, 30 of 45 responders relapsed, six of them after HSCT. Most relapses were of CD22^−^negative/diminished disease. Twenty-one patients were alive at a median follow-up of 9.7 months; 11 of these were in remission, three of whom had received additional therapy for a post-infusion relapse. One patient had ongoing CR >3.5 years post-infusion. Of interest, nine patients received a second infusion: six for CD22^+^ relapse after achieving CR and three for limited CAR T-cell expansion after first infusion. With intensified 4-day lymphodepletion (fludarabine/cyclophosphamide), three of five (60%) patients responded to a second infusion compared to one of four (25%) following 3-day lymphodepletion.

## Commercially Available CAR-T Products

As of September 2021, tisagenlecleucel was still the only FDA- and EMA-approved CAR-T for paediatric patients and AYAs with R/R BCP-ALL. However, and for the sake of completeness, several other CAR-T products have reached market authorisation for indications other than BCP-ALL in adults (Table 2): axicabtagene ciloleucel (Kite Pharma), brexucabtagene autoleucel (Kite Pharma), lisocabtagene maraleucel (Juno Therapeutics), and idecabtagene vicleucel (Bluebird Bio/BMS) (42–49). Some of these products are currently under investigation for their efficacy and safety in BCP-ALL in paediatric patients and AYA.

**Table 2:** CAR-T products with approved market authorisation (by July 2021).

**Drug (company, tradename)**	**CAR construct development**	**CAR design, transduction**	**Current approved indications**	**Year of approval**	**Landmark study**
Tisagenlecleucel^*^ (Novartis, KYMRIAH®)	Children's Hospital of Philadelphia / University of Pennsylvania	CD19 – 4-1BB, Lentivirus	Third-line BCP-ALL <26 years	2017 FDA 2018 EMA	ELIANA (12)
			Third-line PMBCL and DLBCL >18 years	2018 FDA+EMA	JULIET (42)
Axicabtagene ciloleucel^†^ (Kite, YESCARTA®)	National Cancer Institute / Memorial Sloan Kettering Cancer Centre	CD19 – CD28, Retrovirus	Third-line PMBCL and DLBCL >18 years	2017 FDA 2018 EMA	ZUMA-1 (43, 44)
			Third-line follicular lymphoma >18 years	2021 FDA	ZUMA-5 (45)
Brexucabtagene autoleucel (Kite, TECARTUS™)	National Cancer Institute / Memorial Sloan Kettering Cancer Centre	CD19 – CD28, Retrovirus, T-cell enrichment	R/R MCL	2020 FDA 2021 EMA	ZUMA-2 (46)
Lisocabtagene maraleucel (Juno, Breyanzi®)	Seattle group	CD19 – 4-1BB, Lentivirus, CD4/CD8 1:1	Third-line PMBCL, DLBCL, and follicular lymphoma >18 years	2020 EMA 2021 FDA	TRANSCEND (47)
Idecabtagene vicleucel (Bluebird/BMS, ABECMA®)	Bluebird	BCMA – 4-1BB, Lentivirus	Fourth-line multiple myeloma	2021 EMA+FDA	KarMMa (48, 49)

* *Approved for paediatric/AYA BCP-ALL*.

† *Currently under investigation in clinical trials for paediatric/AYA BCP-ALL*.

*AYA, adolescents and young adults; BCP-ALL, B-cell precursor acute lymphoblastic leukaemia; BCMA, B-cell maturation antigen; CAR-T, chimeric antigen receptor T-cell therapy; DLBCL, diffuse large B-cell lymphoma; EMA, European Medicines Agency; FDA, Food and Drug Administration (US); PMBCL, primary mediastinal large B-cell lymphoma; R/R, relapsed or refractory*.

## Factors Influencing Long-Term Efficacy

In recent years, important efforts have been devoted to the development and optimization of CAR T cells redirected against BCP-ALL. Particular attention is given to augment the duration of remission, target new disease subtypes (e.g., infant ALL) and decrease toxicity.

Currently available data on CAR-T in paediatric BCP-ALL point towards several pre-infusion factors that affect the long-term anti-leukaemic efficacy of a CAR-T infusion and thereby the decision of whether or not to consolidate by HSCT. In general, product-related attributes, patient-inherent factors and pre-infusion therapies (e.g., inotuzumab ozogamicin, blinatumomab and lymphodepletion) may all impact on the efficacy and persistence of CAR T cells, as summarised in the next section.

### CAR Design

Results obtained in early clinical studies with so-called first-generation CD19 CAR-T, which contained the ζ chain of the CD3/T-cell receptor (TCR) complex as the only signalling domain (Figure 1A), proved the feasibility of the CAR approach but could not demonstrate objective anti-tumour effects or the persistence of cells after infusion [for a review, see Boyiadzis et al. (50)]. Therefore, second-generation CARs were designed and investigated to incorporate costimulatory endo-domains, mainly CD28 or 4-1BB. These second-generation CAR T cells exhibit less T-cell anergy, have potent anti-tumour activity, secrete significant amounts of cytokines and enhance cell persistence *in vivo*.

Early results of the clinical trials using these CD19 CARs demonstrated a prolonged persistence of constructs encoding the 4-1BB costimulatory domain compared with those incorporating the CD28 costimulatory domain (13, 18, 41, 51–53). In 2015, Long et al. (54) revealed the different molecular impacts of these two costimulatory molecules and showed that CD28 can augment whereas 4-1BB reduces T-cell exhaustion and thereby induces a longer persistence of CAR T cells. Their analyses together with previous reports also underline that the three-dimensional design of the CAR is crucial, if not essential, for correct, more physiological and potent T-cell activity. In fact, not only the costimulatory molecules impact on functionality: the hinge, transmembrane domain and linker also influence it deeply; thus, when CARs without identical hinge and transmembrane domains were compared, differences in CAR T-cell function could be attributed to variations in the hinge and transmembrane domain rather than to differences between the activity of the CD28 and 4-1BB costimulatory domains (55–58). Later in 2018, Quintarelli et al. (59) demonstrated that these effects can be modulated by the administration of IL-7/IL-15 to the T-cell culture and depend on the three-dimensional CAR conformation and scFv used.

Recent meta-analyses of CD19 CAR-T clinical trials did not find statistically significant differences in long-term efficacy (e.g., 1-year PFS) between CD19 CAR T cells containing a CD28 or 4-1BB costimulatory domain (10, 60); however, the analysis was limited by the inclusion of third and fourth generation CARs and confounding was introduced by substantial imbalances between groups in the use of consolidative HSCT, ranging from 0 to 33% (61). However, a difference was observed in the ability to induce MRD-negative remission post-infusion in favour of CAR T cells containing a 4-1BB costimulatory domain (60). Rates of cytokine release syndrome (CRS) varied across trials in the meta-analysis, with no clear association depending on whether a CD28- or a 4-1BB-containing CAR was used (60). Neurotoxicity (immune-effector-cell–associated neurotoxicity syndrome: ICANS) of grade ≥3 did not differ between CD28- and 4-1BB CARs in ALL trials (60).

Third-generation CARs combine costimulatory domains (Figure 1A), but very limited data on their use in BCP-ALL exist. A Phase I/IIa clinical study by Enblad et al. explored the possibility to improve the persistence and activity of CAR T cells using a third-generation CD19 CAR coding CD28 and 4-1BB costimulatory molecules (62). Two of four ALL patients responded. Interestingly, in a Phase I clinical trial in R/R CD19^+^ adult B-cell non-Hodgkin lymphoma (NHL), two different cellular products were simultaneously infused in each patient: one transduced with a second-generation CD19 CAR containing one costimulatory domain (CD28) and another with a third-generation CD19 CAR encoding CD28 and 4-1BB costimulatory domains (NCT01853631). Cells containing the third-generation CAR had superior expansion and longer persistence than did cells containing the second-generation CAR. This difference was most pronounced in patients with low disease burden at infusion and few normal circulating CD19^+^ B cells, a group in which the second-generation CD19 CAR T cells had limited expansion and persistence. As of now, in the very limited number of BCP-ALL patients treated with third- (62) or fourth-generation (63–66) CAR T cells (mainly in Phase I studies), 1-year PFS was substantially lower than that observed with single 4-1BB or CD28 costimulatory domain constructs. However, these comparisons might be biassed by the very limited number of patients analysed as well as differences in the inclusion criteria, manufacturing success, manufacturing time and preconditioning between studies. At the time of writing, no trial of third- or fourth-generation CAR-T in paediatric BCP-ALL was ongoing.

Several preclinical and clinical studies have underlined that one potential mechanism of CAR-T failure is the presence of an immunosuppressive tumour microenvironment, which poses a significant challenge to the efficacy of CAR-T in BCP-ALL and other malignancies (67–71). To overcome the hostile tumour microenvironment, “armoured CAR” constructs (fourth-generation CARs) are under development, which aim to protect and improve the persistence and efficacy of the CAR T cells (Figure 1B) (72–76).

Due to the strict lineage restriction of CD19 to the B-cell compartment, this antigen has until now been the most attractive target in BCP-ALL. As summarised in Table 3, different scFvs derived either from the murine FMC63, SJ25C1 or other antibodies or humanised monoclonal antibodies targeting CD19 have been explored by different groups (11–13, 16, 18, 22, 25, 29, 30, 41, 52, 62, 77–93, 95–108). Even though most clinical trials used a FMC63 scFv, a recent extensive meta-analysis revealed no statistical difference between different scFvs in terms of long-term efficacy (60). To reduce ICANS and CRS and to diminish T-cell exhaustion, Ghorashian et al. (25) designed and investigated a low-affinity CD19.scFv (CAT CD19 CAR-T, Figure 1A), as discussed above. Lastly, the strategy of humanised scFvs is being pursued to avoid the activation of the patient's immune system against murine parts of the CAR and subsequent rejection of the cells and short-term persistence (109).

**Table 3:** Overview of clinical trials for B-cell malignancies using CAR technology.

**Institution**	**Trial ID (disease)**	**Cohort age**	**Target**	**scFv (clone)**	**Spacer**	**Trans-membrane domain**	**Construct**	**Cell origin**	**Trans-duction platform**	**References**
**Autologous T cells**										
Baylor College of Medicine	NCT01853631 (B, C, N)	P/A	CD19	FMC63	CH2-CH3	CD28	CD28. 4-1BB.CD3ζ	Auto T cells	Retroviral	(11)
Baylor College of Medicine	NR	NR	CD19	FMC63	CH2-CH3	CD4	CD3ζ	Auto T cells	Retroviral	(77)
Baylor College of Medicine	NR	NR	CD19	FMC63	CH2-CH3	CD28	CD28.CD3ζ	Auto T cells	Retroviral	–
Bambino Gesù Children's Hospital	NCT03373071 (B, N)	P/yA	CD19	FMC63	CD8	CD8	4-1BB.CD3ζ+iC9	Auto T cells	Retroviral	–
City of Hope	BB-IND-11411 (N)	A	CD19	FMC63	CH2-CH3	CD4	CD3ζ	Auto T cells	Electro-poration	–
Fred Hutchinson Cancer Research Centre	NCT01865617 (B, C, N)	yA/A	CD19	FMC63	IgG4	CD28	4-1BB.CD3ζ+EGFR	Auto T cells	Lentiviral	(39)
Guangdong Provincial People's Hospital	NCT02822326 (B)	P/yA/A	CD19	FMC63	NR	CD28	CD28.CD3ζ+TLR2	Auto T cells	Lentiviral	(78)
Hebei Senlang Biotechnology	NCT02963038 (B, N)	P/yA/A	CD19	FMC63	NR	NR	CD28. 4-1BB.CD3ζ+EGFR	Auto T cells	Lentiviral	(79)
Kite, A Gilead Company	NCT02614066 (B)	yA/A	CD19	FMC63	CD28	CD28	CD28.CD3ζ	Auto T cells	Retroviral	(80)
MD Anderson Cancer Centre	NCT01497184 (B, C, N)	P/yA/A	CD19	FMC63	NR	NR	CD28.CD3ζ	Auto T cells	Electro-poration	–
Memorial Sloan Kettering Cancer Centre	NCT01044069 (B-, C)	yA/A	CD19	SJ25C1	CD28	CD28	CD28.CD3ζ	Auto T cells	Retroviral	(41)
Memorial Sloan Kettering Cancer Centre	NCT01860937 (B)	P/yA	CD19	SJ25C1	CD28	CD28	CD28.CD3ζ	Auto T cells	Retroviral	(16)
National Cancer Institute	NCT00924326 (N)	yA/A	CD19	FMC63	CD28	CD28	CD28.CD3ζ	Auto T cells	Retroviral	(52, 81)
National Cancer Institute	NCT01593696 (B, N)	P/yA	CD19	FMC63	CD28	CD28	CD28.CD3ζ	Auto T cells	Retroviral	(18)
Seattle Children's Hospital	NCT02028455 (B)	P/yA	CD19	FMC63	NR	NR	4-1BB.CD3ζ+EGFR	Auto T cells	Lentiviral	(82)
Sheba Medical Centre	NCT02772198 (B, N)	P/yA/A	CD19	FMC63	CD28	CD28	CD28.CD3ζ	Auto T cells	Retroviral	(83)
Southwest Hospital	NCT02349698 (B, C, N, H)	P/yA/A	CD19	Humanised	CD8	CD8	4-1BB.CD3ζ	Auto T cells	Lentiviral	(84, 85)
University College London	NCT02443831 (B, N)	P/yA	CD19	CAT	CD8	CD8	4-1BB.CD3ζ	Auto T cells	Lentiviral	(25)
University of Pennsylvania	NCT01029366 (B, C, N)	yA/A	CD19	FMC63	CD8	CD8	4-1BB.CD3ζ	Auto T cells	Lentiviral	(22, 86)
University of Pennsylvania	NCT01626495 (B, C, N, H)	P/yA	CD19	FMC63	CD8	CD8	4-1BB.CD3ζ	Auto T cells	Lentiviral	(13)
University of Pennsylvania	NCT02374333 (B, N)	P/yA	CD19	Humanised	CD8	CD8	4-1BB.CD3ζ	Auto T cells	Lentiviral	(87)
University of Pennsylvania	NCT02435849 (B)	P/yA	CD19	FMC63	CD8	CD8	4-1BB.CD3ζ	Auto T cells	Lentiviral	(12)
Uppsala University	NCT02132624 (B, C, N, H)	yA/A	CD19	NR	CH2-CH3	CD28	CD28. 4-1BB.CD3ζ	Auto T cells	Retroviral	(62)
Wuhan Sian Medical Technology Co.	NCT02965092 (B, N, H)	P/yA/A	CD19	NR	NR	NR	4-1BB.CD3ζ	Auto T cells	Lentiviral	(88)
Xuzhou Medical University	NCT02782351 (C)	P/yA/A	CD19	Humanised	CD8	CD8	4-1BB.CD3ζ+EGFR	Auto T cells	Lentiviral	(89)
Zhejiang University	ChiCTR-OCC-15007008 (B, N, H)	P/yA/A	CD19	FMC63	NR	NR	4-1BB.CD3ζ	Auto T cells	Lentiviral	(90)
Hospital Clínic/ Hospital Sant Joan de Déu de Barcelona	NCT03144583 (B, C, N)	P/yA/A	CD19	A3B1	CD8	CD8	4-1BB.CD3ζ	Auto T cells	Lentiviral	(91, 92)
Chinese PLA General Hospital	NCT03097770 (B, C, N)	P/yA/A	CD19/CD20	FMC63+Leu16	CD8	CD8	4-1BB.CD3ζ	Auto T cells	Lentiviral	(93)
Medical College of Wisconsin	NCT03019055 (C, N)	yA/A	CD19/CD20	NR	NR	NR	4-1BB.CD3ζ	Auto T cells	Lentiviral	(94)
Chinese PLA General Hospital	NCT03185494 (B, C, N)	P/yA/A	CD19/CD22	FMC63+m971	NR	CD8	4-1BB.CD3ζ	Auto T cells	Lentiviral	(95)
Hebei Yanda Ludaopei Hospital	NCT04129099 (B)	P/yA/A	CD19/CD22	FMC63+m971	NR	CD8	4-1BB.CD3ζ	Auto T cells	Lentiviral	(96)
City of Hope	BB-IND-8513 (N)	A	CD20	Leu-16	CH2-CH3	CD4	CD3ζ	Auto T cells	Electro-poration	(97)
Beijing Boren Hospital	NR	NR	CD22	Humanised	NR	CD8	4-1BB.CD3ζ	Auto T cells	Lentiviral	(98)
National Cancer Institute	NCT02315612 (B, N)	P/yA/A	CD22	Humanised	NR	CD8	4-1BB.CD3ζ	Auto T cells	Retroviral	(29)
Baylor College of Medicine	NCT00881920 (C, N, MM)	yA/A	κ light chain	FMC63	CH2-CH3	CD28	CD28.CD3ζ	Auto T cells	Retroviral	(99)
**Allogenic T cells**										
Children's Hospital of Fudan University	NCT04173988 (B)	P	CD19	NR	NR	NR	NR	Allo T cells	Lentiviral	–
Chinese PLA General Hospital	NCT01864889 (B-, C, N)	A	CD19	HM852952	CD8	CD8	4-1BB.CD3ζ	Allo T cells	Lentiviral	(100)
Institut de Recherches Internationales Servier	NCT02808442 (B)	yA/A	CD19	NR	NR	NR	4-1BB.CD3ζ+ΔCD20	Allo T cells (αTCR/CD52 depleted)	Lentiviral	(101)
MD Anderson Cancer Centre	NCT00968760 (N)	yA/A	CD19	FMC63	NR	NR	CD28.CD3ζ	Allo T cells	Electro-poration	(102)
National Cancer Institute	NCT01087294 (B, N, H)	yA/A	CD19	FMC63	CD28	CD28	CD28.CD3ζ	Allo T cells	Retroviral	(103)
Peking University	NCT03050190 (B malignancy)	P/yA/A	CD19	FMC63	NR	NR	CD28.CD27.CD3ζ+IC9	Allo T cells	Lentiviral	(104)
Chinese PLA General Hospital	NCT03398967 (B, C, N, H)	P/yA/A	CD19/ CD20 or CD22	4G7	NR	NR	4-1BB.CD3ζ+ΔCD20	Allo T cells (αTCR/CD52 depleted)	Lentiviral	(105)
Baylor College of Medicine	NCT00840853 (B, C, N)	P/yA/A	CD19+ Tri specific virus	FMC63	CH2-CH3	CD28	CD28.CD3ζ	Allo T cells	Retroviral	(106)
Precision BioSciences	NCT04030195 (C, N)	yA/A	CD20	NR	NR	NR	NR	Allo T cells	NR	–
The First Affiliated Hospital with Nanjing Medical University	NCT04176913 (N)	yA/A	CD20	NR	NR	NR	NR	Allo T cells	NR	–
Cellectis S.A.	NCT04150497 (B)	P/yA/A	CD22	NR	NR	NR	4-1BB.CD3ζ	Allo T cells (αTCR/CD52 depleted)	Lentiviral	(107)
**NK cells**										
Fate Therapeutics	NCT04245722 (C, N)	yA/A	CD19	NR	NR	NR	NR	NK cells (iPSC)	NR	–
MD Anderson Cancer Centre	NCT03056339 (B, C, N)	P/yA/A	CD19	FMC63	CD28	CD28	CD28.CD3ζ+IL15	NK cells (cord blood)	Retroviral	(108)

*A, Adult; Allo, allogeneic; Auto, autologous; B, B-cell precursor acute lymphoblastic leukaemia; CAR, chimeric antigen receptor; C, chronic lymphoblastic leukaemia; H, Hodgkin lymphoma; IgG4, immunoglobulin 4; iPSC, induced pluripotent stem cells; MM, multiple myeloma; N, non-Hodgkin lymphoma; NK, natural killer; NR, not reported; P, Paediatric; yA: young adult*.

Some groups have focused on B-cell targets other than CD19, e.g., CD20 (94, 110) and CD22 (Figure 1C). As discussed in a previous section, Shah et al. recently reported the results of a clinical trial exploring the efficacy of CD22 CAR-T encoding 4-1BB as the costimulatory molecule in patients who failed treatment with a CD19 CAR-T (NCT02315612) (30).

To avoid tumour escape mechanisms by antigen loss (111–113), several groups are now investigating the use of bispecific CARs to target BCP-ALL (CD19/CD20 and CD19/CD22) (94, 114, 115) (Figure 1D). Until now, no validated data have been obtained in paediatric patients or AYA with BCP-ALL to prove the safety or superiority in terms of the long-term outcome of targeting another antigen in addition to CD19; however, data in adult ALL and lymphoma have emerged (94, 115). In a Phase I dose-escalation study carried out by Shah et al. (94), the authors demonstrated that in adult B-cell NHL and chronic lymphocytic leukaemia, bispecific CD19/CD20 CAR T cells were able to prevent antigen loss and achieve 64% CR and 18% partial response (PR) at day 28. The ORR was 100% (92% CR and 8% PR) in patients who received the final target dose of 2.5 × 10^6^ of non-cryopreserved CAR T cells/kg (94). No CD19^−^ relapses were observed, demonstrating that the bispecific construct avoided immunological pressure on tumour cells. In contrast, in a Phase I study by Spiegel et al. (115), bispecific CD19/CD22 CAR-T in adult B-ALL and large B-cell lymphoma (LBCL) was not able to overcome CD19 antigen loss. Despite a response rate of 100% in B-ALL (CR) and 62% in LBCL (PR/CR) and low toxicity, 50 and 29% of the relapses in the B-ALL and LBCL cohorts, respectively, were CD19^−/*low*^ whereas none were associated with CD22^−/*low*^ disease.

Results of further clinical trials exploring bispecific CARs will elucidate whether such CAR-T cells could provide a better option than single-antigen-targeted CAR T cells to substitute HSCT. Conversely, a new, non-HSCT strategy could also be explored in which patients who are MRD-positive after CD19 CAR-T receive mono- or bispecific CAR T cells targeting antigens other than CD19 using an allogeneic cellular product.

**Figure 3:**
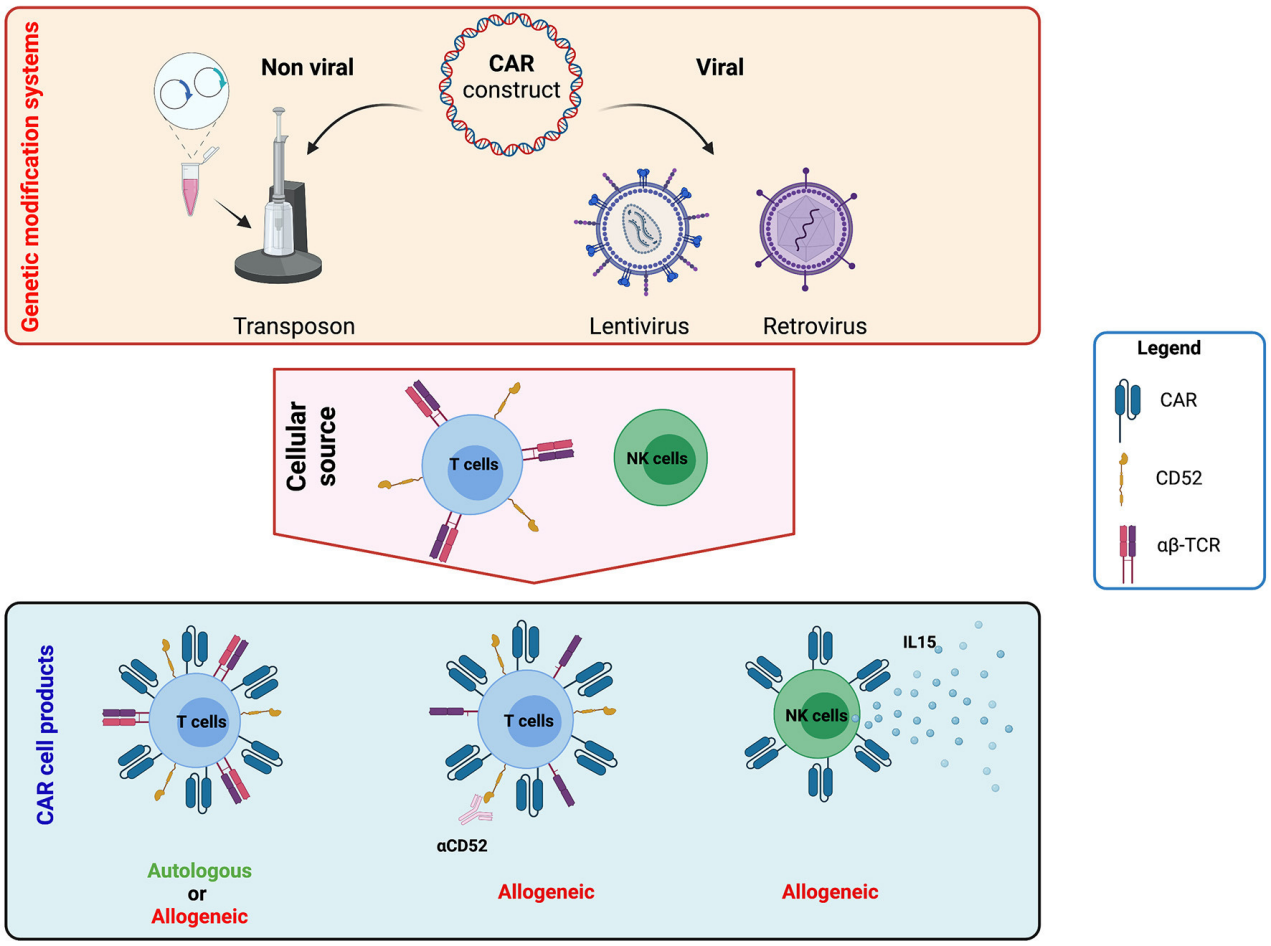
Strategies to generate chimeric antigen receptor (CAR) T and natural killer (NK) cell products. CAR constructs can be generated using viral (lentivirus and retrovirus) and non-viral (transposon system) platforms. The construct can include other elements besides the CAR to increase long-term efficacy and clinical application. For example, it is possible to include the expression of cytokines such as interleukin (IL)-12, IL-15, or IL-18 to improve persistence or gene-editing tools to abrogate the expression of endogenous proteins like T-cell receptor (TCR) elements or CD52. These constructs can then be used to genetically modify either autologous and allogeneic T or NK cells. Image created with BioRender.com.

To introduce the CAR construct into immune effector cells, different platforms for CAR gene transfer have been used, including electroporation (mainly based on the transposon system), as well as lentiviral and retroviral vectors (Figure 3). Based on the data published so far, no difference in clinical outcome has been documented that would reveal the superiority of one of these techniques; however, only transient CAR expression can be achieved after electroporation of plasmids not involving the transposon platform. Even though all three techniques are commonly used to generate autologous and allogeneic clinical-grade CAR products, recent evidence stresses a point of caution regarding the oncogenic potential of transposon systems (piggyBacs) with the first 2 cases of malignant lymphoma derived from CAR genetically modified T cells being described (116, 117). The molecular analysis of these transformed cells revealed a high transgene copy number but no insertion into typical oncogenes. Structural changes such as altered genomic copy number and point mutations unrelated to the insertion sites were also detected. Furthermore, a transcriptome analysis showed transgene promoter-driven upregulation of transcription of surrounding regions despite insulator sequences around the transgene.

Regarding platforms using lentivirus or retrovirus, no evidence of recombination-competent virus or tumour transformation post CAR T cell infusion has been registered so far (118, 119). However, in the first clinical experiences in early 2000's with first-generation retroviral vectors used to stably transduce CD34^+^ stem cells in patients with X-linked severe combined immunodeficiency, T-cell ALL occurred in six out of 20 patients 2–14 years after treatment (120–122). Based on these events, the FDA published guidance for monitoring clinical vector lots, manufactured cell products, and patients post-infusion using biologic or PCR-based testing to detect replication-competent retrovirus (RCR) and lentivirus (RCL). In the two decades since that guidance was published, retroviral packaging cell line and vector designs have minimised the homology between vector and packaging cell sequences and have segregated packaging genes so that the generation of an RCR is extremely unlikely. The segregation of vector components into four plasmids for lentiviral production has similarly ensured that, to date, RCL generation remains only a theoretical possibility (123).

However, the scenario changes and becomes more complicated and, for now, unpredictable when primary T cells undergo several gene modifications, for example lentiviral CAR transduction and TALEN gene editing used to disrupt the T-cell receptor α gene and reduce the incidence of graft-versus-host disease (GvHD). Just recently, the ALLO-501A study (Allogene) was paused due to a chromosomal abnormality detected in a patient with stage IV transformed follicular lymphoma with a *c-myc* rearrangement.

To improve the safety profile of CAR-T and to generate a tool to mitigate/abrogate side effects, gene-based approaches have been developed and validated including: inducible caspase + AP1903, herpes simplex virus thymidine kinase + ganciclovir, truncated epidermal growth factor receptor + cetuximab and CD20 + rituximab (Figure 1E) (124–127).

### CAR-T Product Characteristics

Although registered by the FDA and the EMA as a drug, the characteristics of a CAR-T product are very much different to that of a conventional drug. Variability between individual products may impact outcome. Cell dose, transduction efficiency, cell viability and potency vary between products.

The approved cell dose of tisagenlecleucel is 0.2–5.0 × 10^6^ CAR^+^ T cells per kg body weight for patients <50 kg and 0.1–2.5 × 10^8^ for patients weighing >50 kg. A combined analysis of three tisagenlecleucel trials suggested a positive correlation between the infused cell dose and probability of response in ALL patients (38, 128). Logistic regression analysis showed that a doubling in weight-adjusted dose was associated with a 97% increase in odds of response (38). For patients who weighed >50 kg, the analysis showed a decreased probability of response with doses <2.0 × 10^6^ viable CAR^+^ T cells/kg, and the probability of response plateaued with higher doses (38). However, the studies were not powered to detect dose–response correlations, and few patients were infused with cell doses in the very low range. In US clinical practise, the median cell dose of commercial tisagenlecleucel products reported by Pasquini et al. was lower vs. that in pivotal trials (24). However, all products had cell counts within the approved dosing range and responses were seen at all dose levels with no significant dose–response relationship among patients with ALL. Taken together, an impact of cell dose on clinical outcome cannot be fully excluded. It is advised to target the high end of the dose range and infuse the highest achievable dose for each patient (38).

Low transduction efficiency was an Achilles heel and a limitation in early clinical trials, meaning a higher number of activated and expanded T cells needed to be infused. Furthermore, researchers agreed that the level of transduction was a limiting factor in the comparison of the results from various clinical studies; therefore, current studies are designed to infuse a defined number of genetically modified T cells based on weight or body surface area. However, important results have emerged in recent years highlighting that not only the transduction efficiency plays an important role in the efficacy of the therapy but also the number of molecules/cells (129) and avidity of the CAR (25).

The cell viability of the commercial CAR-T product has been investigated also and compared with published data from the registration trials. The lower acceptable limit for tisagenlecleucel in the post-marketing context is set at 70% viability by the EMA and 80% by the FDA. Products not fulfilling these criteria might be released as out-of-specification (OOS) products based on case-by-case evaluation. Post-marketing registries collect data on patients receiving OOS infusions. No relationship between cell viability <80 or ≥80% in released products and clinical outcome has been demonstrated (24, 130). However, cell viability was lower in real-world products compared with products from the initial trials, the cause of which is currently unclear (24). It will be essential to combine clinical data collected by the treatment sites with the product characteristics to further clarify the impact.

Several efforts have been made to establish new clinical grade strategies to improve *in vivo* CAR T-cell metabolic fitness (131) and thereby persistence. Weber et al. (132) described a strategy to transiently block (“rest”) tonic CAR signalling. Induction of a rest state by enforced CAR protein downregulation using a drug-regulatable system or treatment with the multi-kinase inhibitor dasatinib resulted in a memory-like phenotype, wholescale transcriptional and epigenetic reprogramming, and restoration of anti-tumour functionality in exhausted CAR T cells. Alternatively, other groups propose to epigenetically reprogram CAR T cells at the metabolic level during their production phase using short-chain fatty acids and epigenetic therapeutics in order to overcome barriers limiting CAR T-cell effectiveness (particularly the immunosuppressive tumour microenvironment) and to boost CAR T cells in terms of long-term efficacy (131, 133–135).

### CAR T-Cell Expansion and Area Under the Curve

As a living drug, CAR T cells undergo expansion and persist *in vivo*, which determine the overall CAR T-cell exposure in an individual patient, e.g., during the first 28 days following infusion (AUC_d1−28_). In a study by Mueller et al. (38) combining tisagenlecleucel pharmacodynamic data from ELIANA and ENSIGN, responders had a significantly higher C_max_ (maximum [peak] expansion of tisagenlecleucel) and AUC_d1−28_ compared with non-responders. Patients who relapsed <6 months after infusion had a rapid loss of tisagenlecleucel compared with patients with EFS ≥6 months. CD19^+^ relapses were associated with lower expansion and more rapid loss of transgene expression than that seen in patients with a sustained response. Patients with a CD19^−^ relapse had transgene levels comparable with those of patients with sustained responses.

### Source of T Cells

#### Autologous vs. Donor-Derived Starting Material in a Post-transplant Setting

Outcomes for paediatric patients and AYAs with ALL relapse after HSCT remain poor (136). While CD19 CAR-T offers promising early remission rates, long-term disease control is achieved in <50% of patients and is especially poor when relapse occurs soon after HSCT. In patients who receive CAR-T after a recent HSCT, the T cells collected for CAR T-cell manufacturing are derived from the allograft. There is evidence that, shortly after HSCT, such engrafted T cells might not work well as “autologous” starting material (18), for reasons including exposure to ATG/Campath during conditioning, recent GvHD prophylaxis and/or GvHD treatment or qualitative impairment due to recent engraftment in the recipient. Therefore, when CAR T cells are administered after an allogeneic HSCT, the graft would also allow for the use of “healthy” T cells harvested directly from the donor, which might be better starting material for CAR T-cell generation. A search on ClinicalTrial.gov revealed two ongoing trials using donor-derived CD19 CAR T cells after HSCT (NCT02050347 and NCT01430390). While both trials will administer CD19 CAR T cells harbouring CD28 as the costimulatory domain, only one will use Epstein-Barr virus-specific T cells as the starting material to reduce the risk of GvHD.

Few data are available addressing the GvHD risk associated with infusion of donor-derived allogeneic CAR T cells. The first study using donor-derived multi-virus-specific CD19 CAR T cells was published by Cruz et al. (106). The idea was to reduce the alloreactive potential of donor-derived T cells by selecting and expanding T cells with an endogenous virus-specific TCR, which, due to their TCR specificity and experienced phenotype, would both reduce the risk of GvHD and promote CAR T-cell persistence. While no GvHD or CRS was seen, the CAR T cells indeed expanded upon viral infection or reactivation; interestingly, in this study the CAR T-cell expansion did not cause BCA, suggesting impaired CAR T-cell efficacy when activated through endogenous TCR. The first report using donor-derived non-virus-specific allogeneic CAR T cells was published by Kochenderfer et al. describing results from 10 patients with relapsed B-cell malignancies following HSCT (103). None of the patients receiving donor-derived CD19 CAR T cells developed GvHD. However, patients did not receive lymphodepletion before CAR T-cell infusion and the response rate was very low, with only three patients responding. Three years later, in an updated report of this trial describing the results in 20 patients, still none had experienced GvHD (137). A recently published report from Zhang et al. described results from 43 patients with a B-cell malignancy relapsing after HSCT and treated with donor-derived CD19 CAR T cells from human leukocyte antigen (HLA)-identical siblings or HLA-haplotype-matched relatives (138). While CRS and response rates were quite high (88 and 79%, respectively), only two patients developed grade ≥2 acute GvHD. This study suggests that donor-derived CD19 CAR-T is safe and effective and might be a treatment option for patients relapsing early after HSCT.

#### Third-Party CAR Cells

The use of third-party immune effector cells as the starting material derived from, for example, natural killer (NK) cells (NCT03056339, NCT04245722), invariant NK T cells (NCT03774654), γ/δ T cells (139) (NCT04107142), and α/β T cells knocked-out for the TCR α-chain and CD52 (NCT04150497, NCT03398967) represents an attractive and readily available (“universal”) option for all patients whose lymphocytes could not be collected (in time) or for whom autologous production failed. Moreover, the transduction of a leukaemic cell and, as a result, expansion of CD19 CAR-expressing leukaemic blasts post-infusion has been described as a rare event following autologous leukapheresis (140). Different groups are working on the development and validation of allogeneic third-party CAR cell platforms with the aim to overcome some of the main clinical and economical limitations observed using autologous T cells, including the challenges of leukapheresis and *ad hoc* manufacturing.

In a Phase I study evaluating gene-edited universal CD19 CAR T cells, seven children and 14 adults with R/R BCP-ALL were infused (101). The toxicity profile, including CRS, ICANS and cytopenias, and response rates at day 28 were comparable to those of autologous products, and the disruption of the TCR α/β chain locus indeed effectively prevented alloreactivity against the host (acute GvHD). However, the persistence of universal CD19 CAR T cells was short, even after profound lymphodepletion with fludarabine, cyclophosphamide and alemtuzumab, and the cells persisted beyond day 28 in only three patients. Third-party strategies might offer more cost-effective, efficient and accessible CAR therapies; however, their performance in comparison to other CAR T-cell strategies, and the question of third-party CAR T-cells requiring consolidation by HSCT, still need to be investigated in larger clinical trials.

### Patient-Related Factors

#### Age

The ELIANA trial excluded patients <3 years old, but ~6% of patients in the real-world cohorts were <3 years and these patients had overall responses in line with those of older children (24, 37). A meta-analysis by Anagnostou et al. (60) including 953 evaluable patients demonstrated comparable CR rates between adults and children (adults 75.3% and children 80.5%, *p* = 0.24), but significantly better 1-year OS in children vs. adults (69 vs. 53%, respectively; *p* < 0.01). Toxicity of CRS in adults treated with a single-dose of tisagenlecleucel required adaption to split-dose regimens (141). The impact of age, especially in patients <3 and >25 years, on outcome and toxicity will need further exploration, including using real-world data. The ZUMA-3 trial on axicabtagene ciloleucel enrolled 71 adults with R/R BCP-ALL. Age did not have a statistically significant effect on the primary endpoint of CR/CRi (age 18–39 years: 62%; age 40–64 years: 71%; and age ≥65 years: 100%). Moreover, 6-month EFS and 12-month OS were comparable between the different age groups.

#### Tumour Burden at Infusion

A leukaemic blast count in the bone marrow, or other investigations to evaluate leukaemic disease burden just prior to infusion, has not systematically been performed in all trials; some assessed disease burden only at screening, others prior to lymphodepletion. A high blast count just before infusion has been correlated with increased probability of relapse (37) and lower EFS and OS compared with low disease burden (<5% bone marrow blasts) or undetectable disease at infusion (142). Of note, CD19^−^ relapses occurred more frequently in patients infused with higher tumour burden (37, 143). Notably, the PLAT-02 trial demonstrated decreased CAR T-cell persistence in patients with low (<15%) CD19^+^ counts compared with those with counts >15% (26). Similarly, in real-world data reported by Dourthe et al. (37), higher tumour burden, regardless of the cut-off used (>50 or ≥1%), was associated with longer CAR T-cell persistence but an increased risk of CD19^−^ relapse, whereas a low tumour burden correlated with decreased persistence and increased risk of CD19^+^ relapse. Further, systematic data collection on the pre-infusion tumour burden is necessary to fully understand the impact of CD19^+^ load before infusion on persistence and outcomes of CD19 CAR-T.

#### Genetic Subgroups

Another subgroup of patients more susceptible to CD19^−^ relapses are those harbouring a lysine methyltransferase 2A (*KMT2A*, previously known as *MLL*) gene rearrangement (144). In addition, phenotype switch to acute myeloid leukaemia (AML) can lead to antigen escape (145). Studies demonstrated a variable outcome in *KMT2A*-rearranged patients, potentially due to low patient numbers in any single study. Dual antigen targeting or consolidation with HSCT are proposed treatment options to improve EFS in these patients. However, data to support or prove the efficacy of these strategies are currently lacking. For discussion of CAR-T in patients with breakpoint cluster region protein (*BCR)* and tyrosine-protein kinase ABL1 *(ABL1)* gene fusions, see the companion paper in this supplement by Vettenranta et al. Published real-world data do not show differences in CIR, EFS, or OS between patients with or without a high-risk genetic lesion (including *KMT2A* rearrangements and *BCR-ABL1* fusions) (37).

Children with relapsed BCP-ALL and a *TP53* mutation have a dismal prognosis with standard, intensive treatment protocols for relapse (146), including HSCT (147). CD19 CAR-T followed by consolidation with HSCT was associated with a worse prognosis in patients with a *TP53* mutation compared with patients with wildtype *TP53* (148). CD19^−^ relapses occurred in this subgroup despite consolidation with HSCT, suggestive of an outgrowth of refractory CD19^−^ clones present before HSCT (149, 150). Registry studies might identify other genetic subgroups less or more likely to respond to CAR-T.

Primary resistance to CAR-T occurs in 10–20% of paediatric patients and AYA with BCP-ALL. Singh et al. (151) used genome-scale knockout screening to identify mechanisms related to resistance. A decreased expression of the death receptor pathway resulted in reduced activation of CAR T cells. Bulk RNA expression analysis discriminated patients with a higher risk of non-response. If these data are confirmed, this subgroup of patients might benefit from primary HSCT instead of CAR-T.

### Impact of Pre-treatment on Leukapheresis Feasibility and CAR-T Efficacy

Distinctive features of CAR T cells are that they: (1) need to be manufactured; and (2) are living cells. This means that certain drugs impairing the proliferation or survival of T cells [e.g., lympholytic/lymphotoxic chemotherapy, steroids, tyrosine kinase inhibitors, therapies for GvHD (e.g., calcineurin inhibitors) or lympholytic antibodies such as alemtuzumab and ATG] must be avoided not only immediately before or after CAR T-cell infusion (except if required for the management of severe CAR T-cell toxicities) but also before apheresis in order not to harm the starting material (T-cell numbers and quality in the apheresis product). Some manufacturers or protocols have strictly defined wash-out periods for such drugs prior to apheresis and infusion, ranging from few days to several months depending on each drug's mode of action and half-life (152). Since apheresis timing often depends on a CAR T-cell production slot, especially if the manufacturer requires fresh starting material, and patients often need therapy for their rapidly progressive disease, wash-out periods can be challenging. Korell et al. analysed 75 unstimulated leukapheresis products from healthy donors (*n* = 30) and patients with BCP-ALL (*n* = 6) or lymphoma (*n* = 35) (153). They showed that sufficient lymphocyte yields for CAR T-cell production were feasible even for patients with low leukocyte counts. This is in line with findings of Ceppi et al. who reported successful mononuclear cell targets (100% of all collected apheresis products) and CAR T-cell production (94% of all apheresis products) in 102 aphereses from 99 paediatric patients with neuroblastoma (*n* = 19) or BCP-ALL (*n* = 80) independent of blast counts prior to apheresis (154). These studies show that target harvests for starting material for CAR T-cell generation are obtainable even in heavily pre-treated patients and with low lymphocyte and high blast counts.

Of note, Ruella et al. reported on a patient relapsing 9 months after tisagenlecleucel infusion with apparent “CD19-negative” leukaemia. However, meticulous work-up demonstrated that the relapse consisted of clonal B cells aberrantly expressing the anti-CD19 CAR. Here, the CAR gene was unintentionally introduced into a single leukaemic B cell during CAR T-cell manufacturing. The expressed CD19 CAR then bound in *cis* to the CD19 epitope on the cell surface, masking these CD19^+^ CD19-CAR^+^ cells from recognition by tisagenlecleucel (140).

Current leukapheresis guidelines for the manufacturing of tisagenlecleucel suggest a minimum absolute lymphocyte count (ALC) of 500 cells/μL or a CD3^+^ cell count of 150 cells/μL (if ALC is <500 cells/μL) to start apheresis (155). The PLAT-02 study recommended a minimum ALC of ≥100/μL prior to the apheresis (26). There seems to be a range of lymphocyte counts that allow for the collection of appropriate T-cell numbers for a successful manufacturing process. Certainly, a very low ALC will prolong collection times, which might be challenging for the harvesting facility and the patient, especially in patients <3 years of age. The optimal balance between T-cell numbers and T-cell quality still needs to be determined.

Another concern is that B-cell targeting drugs such as blinatumomab and inotuzumab ozogamicin, which nowadays are frequently used in patients with R/R disease, may impair CAR-T. The concerns are that blinatumomab might increase the selection pressure for CD19^−^ escape variants whereas inotuzumab ozogamicin might deplete the normal B-cell compartment and thereby CD19^+^ targets, severely impacting on CAR T-cell expansion, especially if there is low leukaemic burden as is often induced in inotuzumab ozogamicin responders. Dourthe et al. (37) analysed 51 patients with R/R BCP-ALL receiving commercial tisagenlecleucel and revealed that prior administration of blinatumomab was associated with a shorter EFS and reduced OS due to an increased risk of a CD19^−^ relapse. Moreover, a negative impact on outcome was shown with inotuzumab ozogamicin: seven of 11 inotuzumab ozogamicin-treated patients succumbed to disease. However, since six of those seven died from relapse post-infusion, one cannot exclude that aggressive disease rather than pre-treatment caused CAR-T failure. Awaiting B-cell recovery after use of B-cell–targeting drugs and prior to CD19 CAR-T might play an important role for successful expansion and persistence of CAR T cells. Further studies are planned or ongoing to evaluate this theory.

## Predictive Factors For CAR-T Failure

Unless defined *a priori* in a patient's treatment plan, the decision to consolidate CAR-T infusion with HSCT will in most cases be based on post-infusion observations, particularly after infusion of CAR-T products with potential long-term persistence. A crucial question is therefore if and by which means CAR-T failure can be predicted.

### Persistence of B-Cell Aplasia

Although CAR T cells can be quantitatively measured by real-time qPCR (e.g., detection of the tisagenlecleucel transgene DNA) or FCM, most centres use BCA (as an on-target CAR T-cell effect) as a surrogate marker for CAR T-cell activity, and use B-cell recovery as an indirect indication for CAR T-cell contraction or loss. Indeed, pooled data from the ELIANA and ENSIGN trials demonstrated that B-cell recovery occurring <3 or 3–6 months post infusion was associated with a more rapid loss of CAR T cells measured by transgene levels than when BCA was sustained beyond 6 months (38). Moreover, patients who relapsed in <6 months experienced a more rapid loss of CAR T cells compared with patients with events beyond 6 months. The authors hypothesised that a minimum of 6 months of BCA is necessary to prevent CD19^+^ disease recurrence (38).

The probability of maintaining BCA at 6 months after tisagenlecleucel infusion was 83% in the ELIANA trial (12). In a recent paper by Dourthe et al. (37) focusing on the determinants of CD19^+^ vs. CD19^−^ relapses following tisagenlecleucel therapy in a “real-world” cohort, loss of BCA analysed as a time-dependent variable was associated with increased cumulative incidence of CD19^+^ relapse [sub-distribution hazard ratio 21.7, 95% confidence interval (CI) 2.65–177.70, *p* = 0.004] but not of CD19^−^ relapse. The cumulative incidence of BCA loss was 33, 48, and 55% at 3, 6, and 12 months, respectively. The only predictive factor for BCA loss identified by univariate analysis was MRD <1.0% prior to the lymphodepletion (*p* = 0.03).

### Depth of Remission After CAR-T

Most patients who respond to CAR-T infusion, do so early (by day 28) and have MRD-negative bone marrow [58 of 61 patients in ELIANA (12); >99% in the CIBMTR cohort (24)] unless pre-treated with blinatumomab, which was a predictive risk factor for early failure as defined by the absence of CR or detectable MRD (37). Patients who did not achieve MRD-negativity measured by PCR at day 28 had a dismal prognosis, with an increased CIR (37). However, even patients achieving an MRD-negative remission as assessed by FCM or PCR can later relapse. Therefore, an explorative endpoint in ELIANA was the predictive value of MRD measured by next-generation sequencing (NGS) post tisagenlecleucel infusion. So far, data have been shared only in an abstract and poster (156); NGS-MRD post CAR T-cell infusion was more sensitive than FCM-MRD at detecting impending relapse. NGS-MRD-negativity at day 28 predicted superior relapse-free survival 3 years post infusion compared to NGS-MRD positivity at any level (80 vs. 20%, respectively). The predictive value of NGS-MRD-negativity post infusion has also been reported in adults (157).

### Antigen Stability: Antigen Escape and Lineage Switch

Antigen loss is an escape mechanism common to CAR-T and other targeted therapies, regardless of antigen specificity (29, 111–113). Little is known about the factors predicting CD19^−^ relapse after CD19 CAR-T. As already mentioned, a high blast count prior to infusion was associated with a higher CIR of CD19^−^ relapse (37, 143), and might be explained by an increased risk of the stochastic emergence of CD19^−^ clones escaping CAR T-cell immunosurveillance (37). However, other factors such as the inflammatory context of CRS or the use of anti-IL-6 or steroid therapies may also favour emergence of CD19^−^ clones and need further investigation (37). CD19^−^ relapses seem to occur earlier post infusion than do CD19^+^ relapses (37, 143) and can occur in the presence of BCA (37) and functional CAR T cells. No marker or assay is currently available to predict the emergence of such subclones. Therefore, especially in patients pre-exposed to CD19-targeted therapies like blinatumomab, routine search for CD19^−^ subclones both pre and post infusion is recommended and requires an experienced FCM laboratory.

## Strategies For Preventing Leukaemic Relapse Post CAR-T

The short persistence of CAR T cells, emerging increase of CAR T cells with a resting or exhausted phenotype, early B-cell recovery, and persistence or reappearance of leukaemic clones as MRD are signs of CAR T-cell failure and might trigger interventions to re-establish the CAR-T function and prevent frank relapse.

Re-infusions of CD19 CAR T cells have been used in patients with CD19^+^ relapse or early loss of the CAR T cells with the aim to prolong persistence and reduce relapse risk (26, 37, 53). There is scarce and conflicting information about the efficacy of this approach. Gauthier and collaborators (158) from the Seattle group re-infused their own CAR-T product (see section Other Anti-CD19 CAR-T Products with a 4-1BB Costimulatory Domain) in patients with R/R leukaemia and lymphoma. They found re-infusion to be more effective among patients who had received fludarabine in the first lymphodepletion regimen and in those receiving a 1-log higher dose at re-infusion. However, outcomes among patients with ALL were generally poor, with only 21% of patients responding to re-infusion and a median PFS of only 4 months. Due to the short duration of response, consolidation with HSCT was recommended (158, 159).

Maude et al. (23) reported their experience re-infusing 20 BCP-ALL patients with tisagenlecleucel. Three patients received re-infusion for frank CD19^+^ relapse and 17 for poor CAR T cell persistence after initial infusion (including three who had become MRD positive). New remission was achieved in one of three children re-infused for a CD19^+^ relapse. Of the three MRD-positive patients, one progressed, one became MRD-negative, and one had reduced MRD. Re-infusion induced BCA for a second time in one of seven children treated for B-cell recovery, while six of seven children re-infused for CD19^+^ haematogones continued to have BCA 6–21 months later. A systematic study on tisagenlecleucel re-infusion is ongoing (NCT04225676).

Another approach reported from the same group at the CHOP/University of Pennsylvania (109) is the infusion of a humanised CAR construct (huCART19 or CTL119) in attempt to overcome the possibility of an anti-murine immune response. In a pilot trial (NCT02374333), 33 R/R BCP-ALL patients with either partial or no response to prior tisagenlecleucel, CD19^+^ relapse or early B-cell recovery (defined as occurring within 6 months of prior CAR T-cell infusion), were infused with huCART19 (109). The ORR 1 month after infusion was 64% in the re-treatment cohort. At 6 months after re-treatment, the probability of losing huCART19 persistence was 48% and the incidence of B-cell recovery was 58%.

Finally, to improve CAR T-cell expansion, function and persistence, strategies to combine programmed death 1 (PD-1) checkpoint inhibition (e.g., by pembrolizumab or nivolumab) with CAR T-cell infusion have been reported in BCP-ALL (160, 161). In these small cohorts, PD-1 blockade increased and/or prolonged the detection of circulating CAR T cells. Responses were seen in patients who had early B-cell recovery (re-established BCA) and bulky extramedullary disease (partial response or CR). However, PD-1 inhibition had a partial but not durable effect in patients with a poor initial marrow response to CAR-T alone.

## Interplay Between HSCT and CAR-T in all: Friend or Foe?

As discussed in the previous sections, two alternative strategies have emerged on how HSCT and CAR-T might be used.

### CAR-T as a Bridge to Transplant

The first strategy—which is based on shorter-lived CAR T cells—combines CAR-T and HSCT. Here, CAR T cells are used as a bridge to transplant to induce deep remissions in chemotherapy-resistant patients. This approach takes advantage of two highly effective immunological therapies, CAR-T and HSCT, without abandoning allogeneic transplantation which has proven efficacy and remains the standard of care for high-risk BCP-ALL in both the primary and relapse settings (19, 162–171). The major disadvantages of this approach are HSCT-related toxicity, the price of two costly therapies, and the fact that other more readily available bridging agents like blinatumomab might, for such a strategy, be more practical (and less expensive) alternatives to CAR-T. Moreover, this approach is challenging in patients who have been transplanted before and are not eligible for a second HSCT.

To compare such a strategy to current practise and outcomes, a study would need to include two treatment arms—one with CAR-T bridging and one with alternative bridging therapy—both ending in HSCT consolidation. Similar study designs have been used e.g., in trials of blinatumomab for first BCP-ALL relapse (164, 167).

### CAR-T as an Alternative to HSCT

The second strategy, mainly based on CAR T cells with an extended persistence, has the aim to replace HSCT (i.e., to implement CAR-T as a stand-alone treatment). The obvious main advantage of this approach is the avoidance of a toxic HSCT procedure with its associated risks of serious long-term complications in paediatric populations. Disadvantages include long-lasting BCA as an on-target effect of B-cell-targeting CAR-T. This can be handled by immunoglobulin replacement but the long-term effects of a CAR-T-induced BCA on the immune system of children needs further observation. Moreover, CAR-T targeting single antigens, even with persistence, brings the risk of provoking target-negative subclones that could potentially be eliminated by the broader graft-vs.-leukaemia effect of a consolidative HSCT post CAR-T. Alternatively, multi-antigen targeting approaches may overcome tumour escape in a CAR-T stand-alone strategy (172, 173).

To compare a CAR-T stand-alone strategy to the current practise (which includes HSCT), studies would need one treatment arm to end at CAR-T whereas another arm would extend to HSCT, allowing the best available and most appropriate bridging therapy for each arm beforehand.

## Knowledge Gaps in the Use of CAR-T

As extensively exemplified, CAR-T is a multifaceted therapy with broad diversity in terms of the CAR design, pharmacodynamic attributes, long-term performance, and persistence. In addition, the field is rapidly moving forward with new or refined constructs, starting materials, manufacturing optimizations, and novel combinations and overall strategies emerging all the time. The answer of whether CAR-T can replace HSCT or should be a bridge to HSCT will depend on the properties of the specific CAR-T in question.

Current data on CAR-T in BCP-ALL in paediatric patients and AYAs are mainly derived from Phase I and II studies. These studies focused on early responses and safety, generating important data on several products. Still, compared with data derived from HSCT studies, there is insufficient information on key aspects of CAR-T to guide the positioning of CAR-T relative to HSCT, as outlined below.

- **Safety/toxicity**: further long-term studies are needed to follow acute CAR-T toxicities such as ICANS, CRS, and BCA, and also to detect subtle or subacute toxicities that might appear with longer observation times post infusion (27, 174).
- **Efficacy**: more data are needed on the long-term efficacy of CAR-T, especially focusing on therapies given in addition to the initial CAR T-cell infusion (e.g., re-infusions, secondary CAR-T products, immunomodulatory agents such as checkpoint inhibitors, molecular targeted therapies such as tyrosine kinase inhibitors, and consolidative HSCT while patients are still in remission).
- **Cost**: strategies based on sequential therapies (e.g., CAR-T followed by HSCT; HSCT followed by CAR-T; CAR-T followed by CAR-T; CAR-T followed by HSCT followed by CAR-T etc.) challenge healthcare system budgets. The overall costs for cure must be considered. Studies published to date did not fully disclose the overall longitudinal treatment journey of individual patients before definite therapy was applied. Sequential strategies are especially challenging in middle- and low-income countries.

Finally, there is a lack of Phase III studies that robustly compare current standard of care (which includes HSCT) to the CAR-T approaches aimed to replace HSCT. To be able to draw firm conclusions from such studies, the tremendous heterogeneity in previous Phase I/II cohorts regarding stage of BCP-ALL, cytogenetics, pre-treatments and products used need to be minimised or controlled by defining clear study entry criteria, cohorts and endpoints.

## Ongoing and Planned Studies to Close the Gaps

### Further Research on Approved CAR-T Indications

Currently, as of September 2021, tisagenlecleucel is the only CAR-T with market authorisation for paediatric patients and AYAs with BCP-ALL. As discussed above, the approved indications are ≥2 BCP-ALL relapse or a relapse after HSCT (Figure 2). In addition, refractory disease to standard chemotherapy, either in a primary or relapsed setting, is an indication for tisagenlecleucel.

As a post-market requirement applied by the regulatory authorities, data on patients receiving commercial tisagenlecleucel are collected in registries such as the CIBMTR (24), European Society for Blood and Marrow Transplantation (EBMT) registry (175, 176) or national registries (37). These “real-world” databases collecting data on a rapidly increasing number of patients will be valuable (but yet not monitored) resources to begin to evaluate in more detail (retrospectively and prospectively) multiple aspects of CAR-T planning, delivery, and outcome (Table 4).

**Table 4:** Aspects of CAR-T planning, delivery and outcome that could be researched using post-marketing registry data.

**Theme**	**Example topics of research**
Determinants of outcomes	• Disease-specific characteristics prior to CAR-T infusion e.g., age, cytogenetics, timing and site of relapse, previous therapies (including blinatumomab and inotuzumab ozogamicin), and pre-existing toxicities • Choice of bridging therapy • Product-specific variables (apheresis starting material, CAR T-cell dose, manufacturing failures or delays, out-of-specification products)
Long-term efficacy variables (beyond 1-month overall response rate, early event-free survival and overall survival)	• MRD-negativity over time (including by next-generation sequencing) • Lineage switches (*KMT2A*-r/*BCR-ABL1*+ patients) • Persistence of CAR T cells and duration of B-cell aplasia • Incidence, duration and impact of immunoglobulin substitutions • CD19^+^ vs. CD19^−^ relapses: ratio and determinants
Interventions post-infusion	• Consolidative HSCT, analysed as an event and/or study endpoint (“HSCT- and MRD-free survival”) • Role and rate of CAR-T re-infusion • Tyrosine kinase inhibitors or any other BCP-ALL-targeted therapy
Longitudinal follow-up per patient (route to cure)	• Total number of therapies • Sequence of therapies • Length of overall therapy
Cost	• Total costs of BCP-ALL treatment (from diagnosis to cure) • Comparison of CAR-T as a bridge to transplant with other bridging therapies e.g., blinatumomab

*ABL1, tyrosine-protein kinase ABL1; BCP-ALL, B-cell precursor acute lymphoblastic leukaemia; BCR, breakpoint cluster region protein; CAR, chimeric antigen receptor; CAR-T, CAR T-cell therapy; HSCT, haematopoietic stem cell transplantation; KMT2A-r, lysine methyltransferase 2A rearranged*.

In addition, a consistent definition of refractory BCP-ALL is lacking (i.e., MRD persistence vs. non-CR). It important to know how many patients received tisagenlecleucel for refractory BCP-ALL based on MRD persistence, and what the outcomes were for these patients.

### Research in Primary BCP-ALL

The important question of whether CAR-T can circumvent the long-term adverse effects of HSCT is currently not being addressed in a randomised study design in any ALL disease stage. However, the CASSIOPEIA study (NCT03876769), a single arm, multi-centre, Phase II trial, will determine the efficacy and safety of tisagenlecleucel in paediatric patients and AYAs with *de novo* NCI-defined high-risk BCP-ALL who have received a four-drug induction and subsequent consolidation (~3 months of therapy in total) yet remain MRD positive at the end of consolidation, defined as an MRD of >0.01% by centralised FCM assessment (177). Such patients have a dismal prognosis with conventional high-intensive chemotherapy blocks consolidated by HSCT (178, 179), and experience substantial therapy-related toxicity (180). CASSIOPEIA is not randomising HSCT against CAR-T but will have a historic NCI-defined high-risk BCP-ALL cohort [COG study AALL0232 (179)] as the comparator. Tisagenlecleucel is being infused as a definitive therapy; only patients with an early loss of BCA or with MRD re-appearance and who are not responding to an optional CAR T-cell re-infusion will be eligible for additional HSCT. The endpoint of this study is 5-year DFS with secondary malignancy, death or morphological relapse defined as events. After ELIANA, this will be the first study to focus on further expansion of the indications for tisagenlecleucel in BCP-ALL and aims to investigate whether CAR-T can achieve outcomes in primary high-risk BCP-ALL that are comparable to those achieved with standard high-risk block therapies and HSCT but with reduced toxicity.

### Research in First BCP-ALL Relapse

Despite a strong desire by paediatric haemato-oncologists, it has not yet been possible to set up a study with tisagenlecleucel for patients in first relapse. In the 2010 European IntReALL SR protocol (NCT01802814), patients with standard-risk first relapse were stratified to HSCT only if they responded insufficiently to re-induction treatment. In the IntReALL HR protocol (NCT03590171) patients with high-risk characteristics of first relapse were eligible for HSCT provided they entered remission on chemotherapy +/- blinatumomab. Only patients truly refractory to relapse therapy are within the current indication of commercial tisagenlecleucel.

However, studies are being conducted with CAR-T products other than tisagenlecleucel in which patients with a first relapse qualify for CD19 CAR-T. The ZUMA-4 trial (NCT02625480), which includes patients with a first relapse within 18 months of diagnosis, may broaden the CAR-T indication in the future. However, current long-term follow-up data on the brexucabtagene autoleucel product used in ZUMA-4 (CD28.CD3ζ CAR) indicate that consolidative HSCT is mandatory in all responding patients (27).

The TRANSCEND PEDALL study (NCT03743246), after establishing the recommended Phase II dose of the CAR-T product JCAR017, will also include patients with a first relapse and MRD positivity after re-induction therapy. A recent update of the PLAT-02 Phase I/II trial (JCAR017 in R/R BCP-ALL, NCT02028455) demonstrated an advantage of consolidative HSCT vs. watchful waiting with this CAR construct (181).

**Figure 4:**
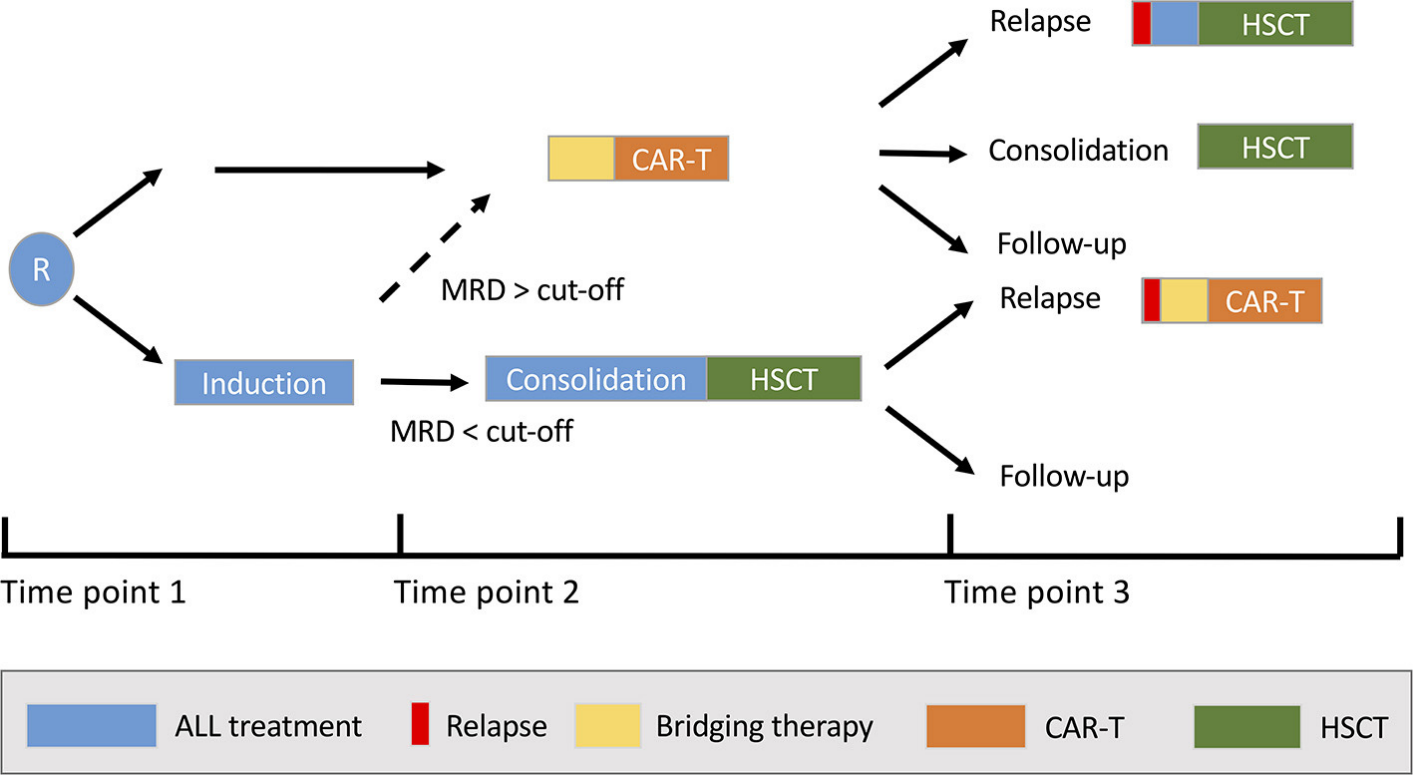
Potential study design for a randomised study comparing chimeric antigen receptor (CAR) T-cell therapy with allogeneic haematopoietic stem cell transplantation (HSCT) in children with a first relapse of B-cell precursor acute lymphoblastic leukaemia. MRD, minimal residual disease; R, randomisation.

To answer the question of whether HSCT can be avoided in a first relapse setting, studies are needed with CAR-T products persisting for a sufficiently long time to serve as a stand-alone therapy. Ideally, these studies should randomise patients at relapse and include all patients currently allocated to HSCT to collect a T-cell apheresis product before the start of any chemotherapy. A proposed design is presented in Figure 4. Patients could be randomised (time point 1 in Figure 4) to either CAR-T or HSCT. In the CAR-T arm, an algorithm of different strategies for bridging would be essential to harmonise chemotherapy or immunotherapy before infusion according to variable prior toxicity and resistance patterns. After CAR-T (time point 3 in Figure 4), a subset of patients would either be primary refractory to CAR-T or relapse despite CAR-T and undergo HSCT. This cohort, although heavily selected, could be compared to historic controls to determine whether prior CAR-T impacts the outcome of HSCT. Children allocated to the HSCT arm would receive induction and consolidation chemotherapy and HSCT. However, as patients with persistent MRD (at a defined cut-off; time point 2 in Figure 4) have a notoriously poor prognosis, these patients would switch arm and be offered CAR-T as salvage treatment. Patients relapsing after HSCT would also be candidates for CAR-T, following the approved indication. A similar design could also be developed for upfront BCP-ALL treatment.

In addition, studies need an algorithm to define for which patients consolidation with HSCT is recommended despite MRD-negativity. Such an algorithm could be based on the different aspects discussed in this review. Factors will include the CAR-T product attributes, the duration of CAR T cell persistence/BCA, depth of remission based on MRD (potentially by NGS), salvage therapy options and genetic high-risk characteristics (e.g., *TP53* mutations) at study inclusion. In the latter group, in which particularly little is known about the long-term efficacy of CAR-T (see section Factors influencing long-term efficacy - patient-related factors - genetic subgroups), it must be thoroughly considered whether CAR-T is indeed the most cost-effective treatment to induce remission or whether HSCT should be mandatory.

Another unanswered question is whether CAR-T in the event of a new BCP-ALL recurrence impacts on DFS after subsequent conventional high-dose chemotherapy followed by TBI-based HSCT.

Ideally, the above studies should be randomised, prospective, and longitudinal. The comparison of outcomes with historic control cohorts is complicated by the recent introduction of novel therapies (e.g., blinatumomab) into standard-of-care relapse protocols. Another complicating factor for study design is the proportion of patients who might crossover between treatment arms: patients randomised to chemotherapy and HSCT might become eligible for CAR-T in the event of an insufficient response (refractory or not achieving MRD-negativity), and, vice versa, patients receiving primary CAR-T therapy might receive HSCT as consolidation in the event of early loss of BCA or MRD reappearance. The DFS of children with an indication for HSCT in CR1 or CR2 and after standard-risk or high-risk salvage induction is not comparable. Therefore, future studies should stratify patients by the indication for HSCT or separate studies should be initiated.

## Discussion

The question of whether CAR-T is a stand-alone therapy or a bridge to transplant cannot generally be answered with the current data. There is a lack of randomised studies comparing approaches with consolidative HSCT vs. approaches in which patients will not proceed to HSCT but are strictly followed for CAR T-cell persistence and MRD remission post-infusion. The trials published to date are heterogeneous in terms of the CAR itself (design, target, and affinity), the CAR-T product attributes, the study population (fraction of patients with post-HSCT relapse at CAR-T study inclusion, genetic subgroups, age groups (e.g., <3 years of age), blast count prior to infusion, and the overall treatment strategy (consolidation by HSCT as part of the protocol).

There seems to be a consensus among researchers that CAR T cells need to persist for a while to be effective as stand-alone therapy; however, the necessary duration of persistence, measured either directly by CAR transgene levels or FCM, or using the duration of BCA as a surrogate marker, is unclear. The “short-lived” CAR-T products are mainly consolidated by HSCT, and very few patients have survived without HSCT. In patients who have received CAR-T products with potential long-term persistence, no definite general recommendation can be made.

**Figure 5:**
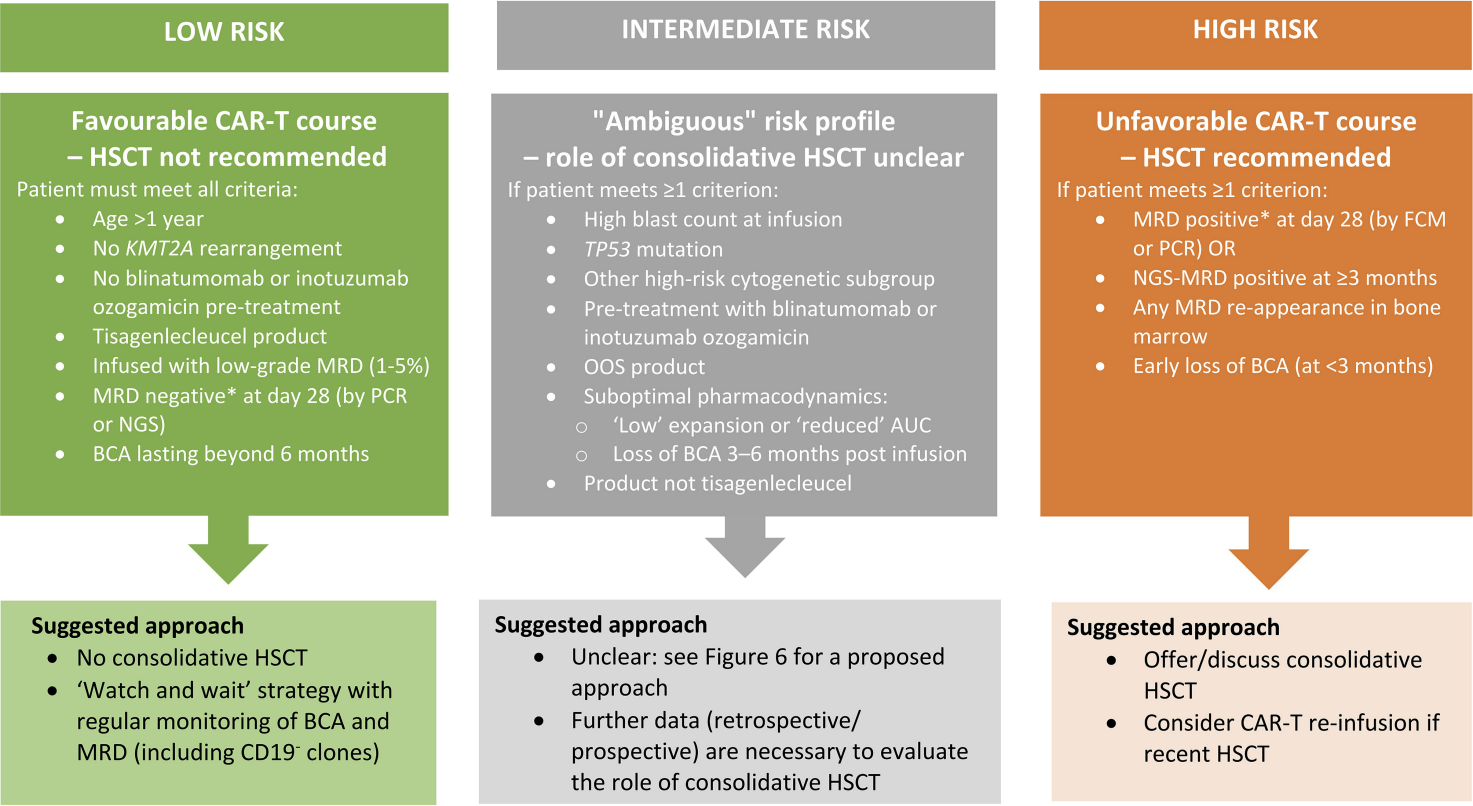
Proposed approach to HSCT consolidation after CAR-T for paediatric patients and AYA with BCP-ALL based on treatment- and disease-related risk factors for relapse. *MRD positivity defined at >0.01%. AUC, area under the curve; AYA, adolescent and young adult; BCA, B-cell aplasia; CAR-T, chimeric antigen receptor T-cell therapy; FCM, flow cytometry; HSCT, haematopoietic stem cell transplantation; KMT2A, lysine methyltransferase 2A; MRD, minimal residual disease; NGS, next-generation sequencing; OOS, out of specification; PCR, polymerase chain reaction.

However, looking at the data on tisagenlecleucel efficacy, there seems to be a subgroup of patients with a very favourable therapy course (a “low-risk group”) in which the chance of cure by CAR-T alone is very high (see Figure 5): age >1 year; no *KMT2A* rearrangement; no blinatumomab or inotuzumab ozogamicin pre-treatment; tisagenlecleucel product; infused in remission but with low level MRD (e.g., bone marrow blast count 1–5%); MRD-negativity at day 28 [by PCR (37) or even better, by NGS (182)]; and BCA lasting >6 months. In such a patient, we suggest a watch-and-wait strategy (with regular monitoring of CD19^−^ clones) without consolidative HSCT.

Conversely, there appears to be a subgroup of patients with an unfavourable course following CAR-T (a “high-risk group”) with a very high chance of treatment failure and, likely, an indication for consolidation by HSCT: MRD positivity at day 28 (by FCM or PCR), NGS-MRD positivity at ≥3 months, or any MRD re-appearance in the bone marrow (measured by any method); and early loss of BCA (<3 months) (Figure 5). Based on current data, these patients should be offered HSCT as further consolidation of MRD-negative remission.

**Figure 6:**
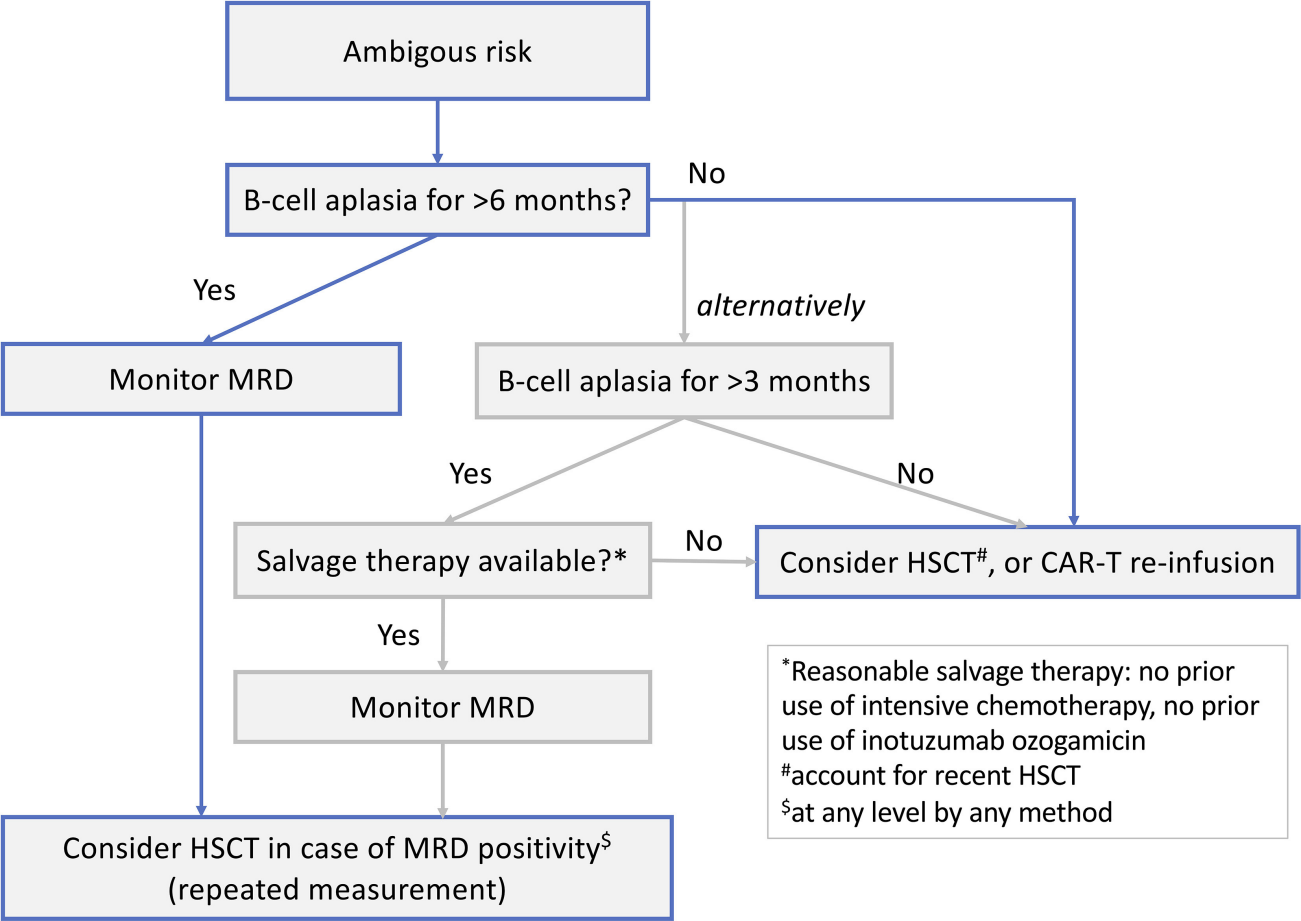
Follow-up guidance after CAR-T for paediatric patients and AYA with BCP-ALL and an “ambiguous risk profile” (see Figure 5 for criteria for an ambiguous risk profile).

For all other patients who may have identified risk factors for long-term CAR-T failure (e.g., high blasts count at infusion, *TP53* mutation, certain high-risk cytogenetic subgroups, pre-treatment with inotuzumab ozogamicin or blinatumomab, OOS products, suboptimal pharmacodynamic parameters such as e.g., “low” expansion or “reduced” AUC, and loss of BCA 3–6 months post infusion), or after infusion of products other than tisagenlecleucel, no firm recommendations can be made on the advantage, timing or clear indication for consolidative HSCT because of a lack of sufficient data. The question of whether this “ambiguous risk group” will profit from HSCT consolidation cannot be answered currently. However, based on our clinical experience with tisagenlecleucel, the decision for or against HSCT in this group may be guided by the length of BCA, other potential salvage options and re-appearance of MRD (Figure 6). Larger cohorts and prospective studies with stringent protocols and endpoints will be necessary (including, for example, standardised measurement of CAR T cells, defined timepoints for MRD, and CAR-T quantification) to define the best treatment strategy for such patients.

## Conclusion

Paediatric patients and AYA with BCP-ALL who are candidates for CAR-T and HSCT represent very rare patient populations. The only way to get valid answers on the overarching questions of when and how to treat high-risk patients with one or other approach is broad, international collaboration on well-defined studies. Fortunately, paediatric oncology already has strong research networks and has a long tradition in cooperative efforts; thus, with additional data support from CAR-T and HSCT registries of the EBMT and CIBMTR and a willingness of companies to support necessary randomised trials, we would be positioned to address these questions altogether. The successful collaboration on the ALL SCTped 2012 FORUM trial, gathering investigators from 119 centres in 32 countries committed to answer one important randomised question, exemplifies what the field can achieve.

## Author Contributions

JB, FC, IC, AK, and SR wrote the review. JB coordinated the writing process. KV, PB, AB, and CP critically revised the drafts. JB, FC, and IC made the figures and tables. All authors contributed to the conception of this review and have made a substantial, direct, and intellectual contribution to the work and approved it for publication.

## Funding

This study received funding from the Parents' initiative for children with leukaemia and solid tumour in Würzburg e.V., Rainbow association for children with leukaemia and solid tumour Main-Tauber e.V., a DKMS—John Hansen Research Grant (DKMS-SLS-JHRG-2021-03), and a Norwegian Children's Cancer Foundation Grant (project 190012). The funders were not involved in the study design, collection, analysis, interpretation of data, the writing of this article, or the decision to submit it for publication.

## Conflict of Interest

JB: has received personal fees, advisory board/steering committee honoraria, and non-financial support from Novartis; and advisory board honoraria from Pfizer, Kite, and Janssen. SR: consultant or advisory role (Novartis, Jazz, Shire/Servier, and Amgen), travel grants (Novartis, Jazz, Shire/Servier, and Amgen), and honoraria (Novartis, Jazz, Shire/Servier, and Amgen). CP: advisory board: Medac, Novartis, Neovii, and Amgen; travel grants, speakers bureau: Amgen, Pfizer, Novartis, Riemser, and Medac. KV: has received personal fees, advisory board/steering committee honoraria, and non-financial support from Novartis. AB: has received personal fees, advisory board/steering committee honoraria, and non-financial support from Novartis; and advisory board honoraria from Kite, Servier, Jazz, and Celgene. The remaining authors declare that the research was conducted in the absence of any commercial or financial relationships that could be construed as a potential conflict of interest.

## Publisher's Note

All claims expressed in this article are solely those of the authors and do not necessarily represent those of their affiliated organizations, or those of the publisher, the editors and the reviewers. Any product that may be evaluated in this article, or claim that may be made by its manufacturer, is not guaranteed or endorsed by the publisher.
